# Supplementary material for: Genome wide association study meta-analysis of neuropathologic lesions of Alzheimer’s disease and related dementias in a multi-site autopsy cohort
Source: PLoS Genet. 2026 Jun 29;22(6):e1012170. doi: 10.1371/journal.pgen.1012170 (PMC13340787; doi:10.1371/journal.pgen.1012170)
Supplement: S1 Text — Text includes results, discussion, and methodology specific to SNP heritability analyses. (DOCX) [file pgen.1012170.s001.docx]

**Supplemental Text**

**Cell and tissue enrichment of SNP heritability.** We used heritability-enrichment methods to assess cell and tissue-specific polygenic effects for neuropathologic changes that is, we assess whether the SNP heritability estimates noted above are concentrated or enriched in specific cell or tissue types. Referent cell types were generated by a genome-wide study of tissue-specific gene expression in humans (GTEx), and a study of immune cell types in mice (ImmGen). Among the GTEx cell types, 7 cell types show significant enrichment (false discover rate, FDR < 5%) with a neuropathologic lesion (**Fig 8; S10, S11**). These include multiple central nervous system (CNS) types implicated with LBD (including substantia nigra), a granulocyte precursor cell with cerebrovascular atherosclerosis severity, and colon sigmoid with presence of CBVD. More broadly, presence of APs was nominally enriched in multiple CNS tissue types, including frontal cortex, hypothalamus, and others. Conversely, NP severity (C score) and NFT severity (B score) both of which share the accumulation of pathologic tau species, heritability tended to show enrichment in genes connected to blood and immune cell types. NP severity showed enrichment with monocytes, phagocytes, macrophages, and others; NFT progression with dendritic cells, macrophages, and others. Presence of cerebrovascular arteriolosclerosis heritability was enriched in adipose tissue types, while severity was enriched in digestive tissue types. Similarly, CAA severity showed enrichment among digestive tissue types. In the assessment of immune cell types, only one showed FDR significant enrichment: LBD heritability enriched in GN.UrAc.PC (myeloid) genes. We also observed nominal enrichment in B cells for NFT, presence of atherosclerosis, and severity of arteriolosclerosis (**Fig S11, Supplemental Tables 12, 13**). NP severity (CERAD) and LBD phenotypes showed nominal enrichment in myeloid cells, while Thal phase heritability enrichment was mostly restricted to stromal cells.

**Discussion**

Tissue enrichment analysis of heritability shows enrichment in CNS (unsurprisingly) but also confirms the relevance of genes common to blood and immune pathways. Cell enrichment analysis suggests a role for myeloid-related genes in AP, but B cells in NFT, suggesting immune/inflammatory response may differ for amyloid and NFT phenotypes. We note that, while these results are consistent with an amyloid/tau/vascular hypothesis for neuronal loss, the cell/tissue enrichment data are fundamentally limited by the gene expression data underlying the models. Particularly, a lack of enrichment in a particular cell type could be because the appropriate stage of development, disease, or risk exposure was not well represented in the referent data.

**Methods**

Cell-specific enrichment of heritability was estimated using LDSC (--ref-ld-chr-cts) with the above noted parameters for heritability. Cell-specific references are derived from human gene expression data (GTEx) and an immune gene dataset derived from mouse (ImmGen) as described in Finucane *et al.* (2018, *Nat Gen*). Cell-types were labeled for plotting according to Finucane *et al.* (2018, *Nat Gen*), supplemental material (Tables 6, 8, and 10). For cell enrichment analyses we utilized the 5% false discovery rate thresholds noted by Finucane *et al*., corresponding to -log10(p-value)=2.75 (*P* < 0.00178) for the GTEx cell types and -log10(p-value)=3.03 (*P* < 0.000933) for ImmGen types.
