# Supplementary material for: Genome wide association study meta-analysis of neuropathologic lesions of Alzheimer’s disease and related dementias in a multi-site autopsy cohort
Source: PLoS Genet. 2026 Jun 29;22(6):e1012170. doi: 10.1371/journal.pgen.1012170 (PMC13340787; doi:10.1371/journal.pgen.1012170)
Supplement: S1 Fig — Spearman correlation of neuropathology phenotypes and relevant covariates. Acronyms and abbreviations: APOE4, count of e4 allele; AAD, age at death; Thal, Thal Phase; Braak, NFT Braak stage; CERAD, CERAD NP score; ADNC, ADNC score (ABC score); ATH, cerebrovascular atherosclerosis; ART, cerebrovascular arteriolosclerosis; CAA, cerebral amyloid angiopathy; LBD, Lewy body disease; INFA, macroinfarcts/lacunes; MICR, microinfarcts; WMR, white matter rarefaction; VBI, vascular brain injury; HS, hippocampal sclerosis; TDP43, TDP-43 proteinopathy. (DOCX) [file pgen.1012170.s002.docx]

## Figure S1: Correlation plot of neuropathology and related variables


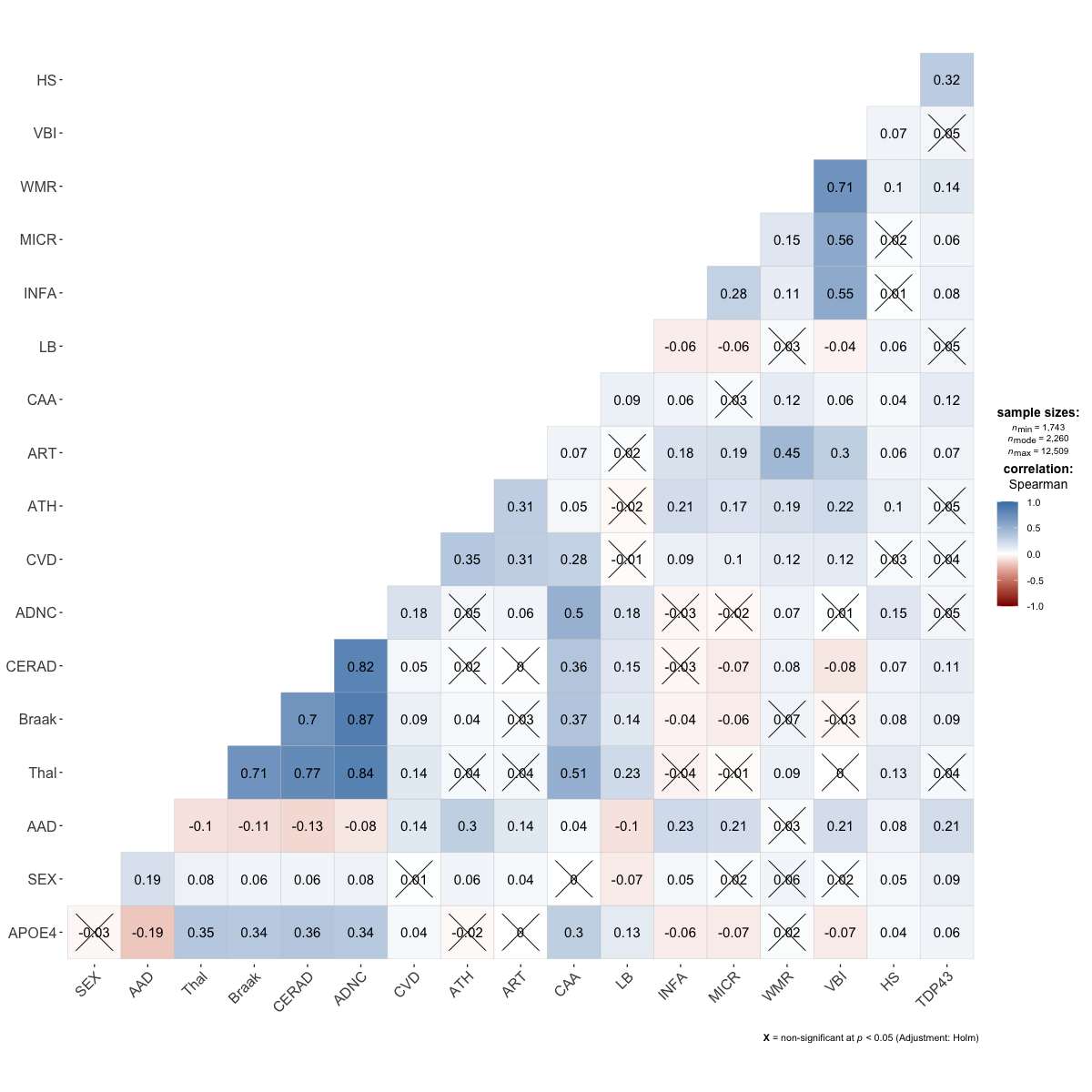


Legend: APOE4, count of e4 allele; AAD, age at death; Thal, Thal Phase; Braak, NFT Braak stage; CERAD, CERAD NP score; ADNC, ADNC score (ABC score); ATH, cerebrovascular atherosclerosis; ART, cerebrovascular arteriolosclerosis; CAA, cerebral amyloid angiopathy; LBD, Lewy body disease; INFA, macroinfarcts/lacunes; MICR, microinfarcts; WMR, white matter rarefaction; VBI, vascular brain injury; HS, hippocampal sclerosis; TDP43, TDP-43 proteinopathy. Crossed cells indicate non-significant correlations.
