# Supplementary material for: Genome wide association study meta-analysis of neuropathologic lesions of Alzheimer’s disease and related dementias in a multi-site autopsy cohort
Source: PLoS Genet. 2026 Jun 29;22(6):e1012170. doi: 10.1371/journal.pgen.1012170 (PMC13340787; doi:10.1371/journal.pgen.1012170)

## Figure S2: QQ plots for genome-wide association analyses

Legend: Quantile-quantile plots for each genome-wide association study. λ denotes the genomic inflation factor for the study.
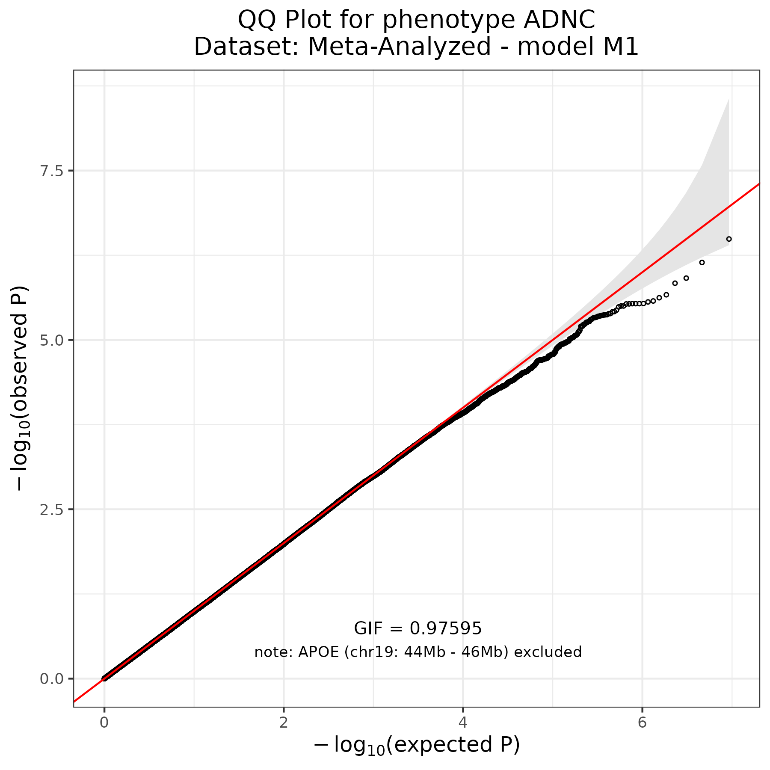

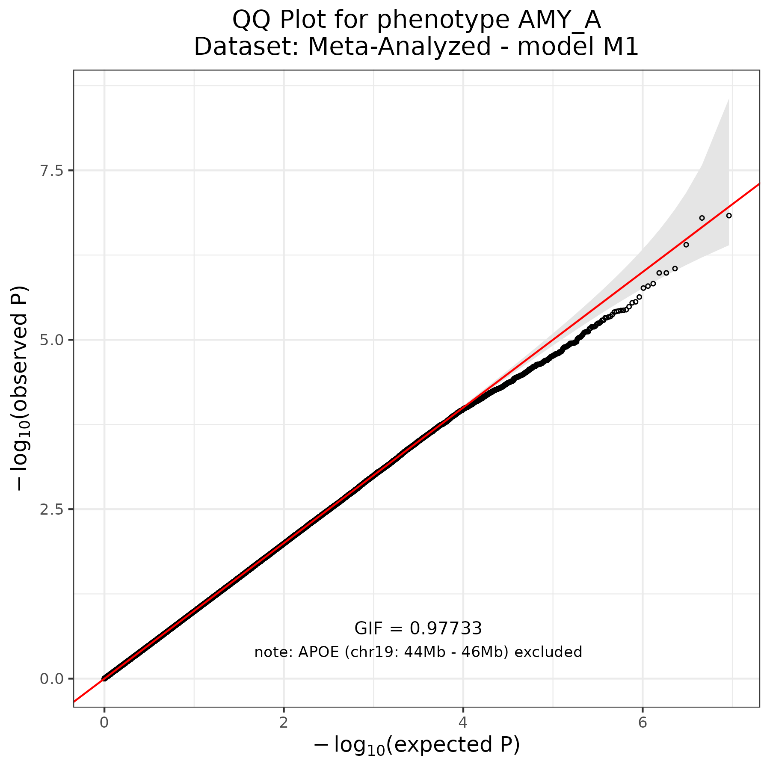

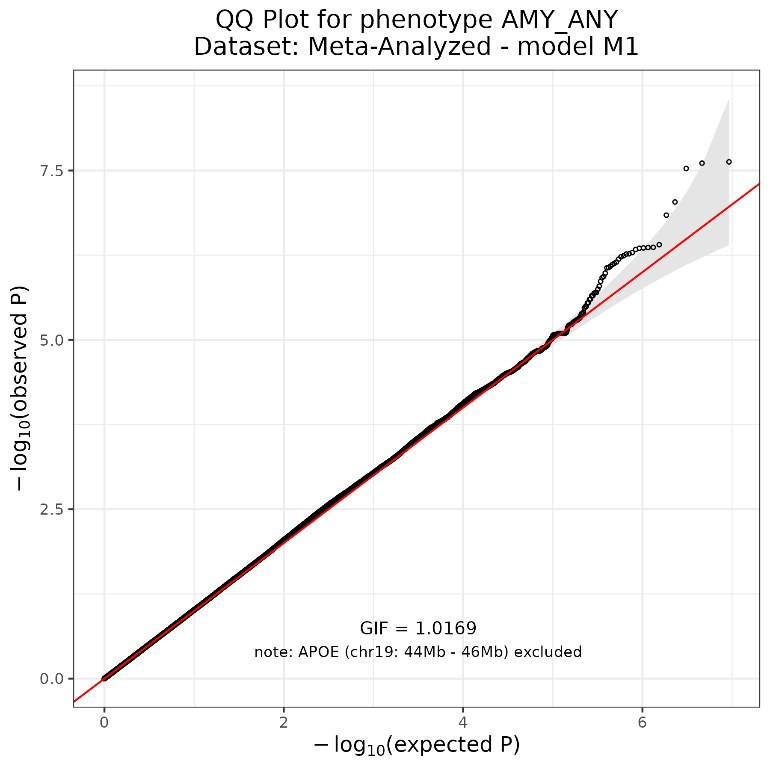

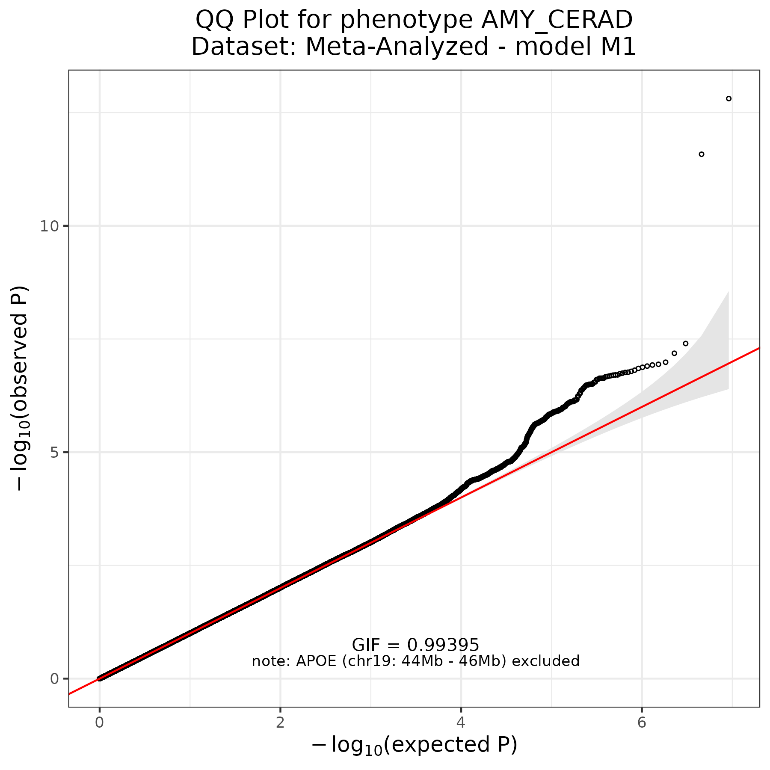

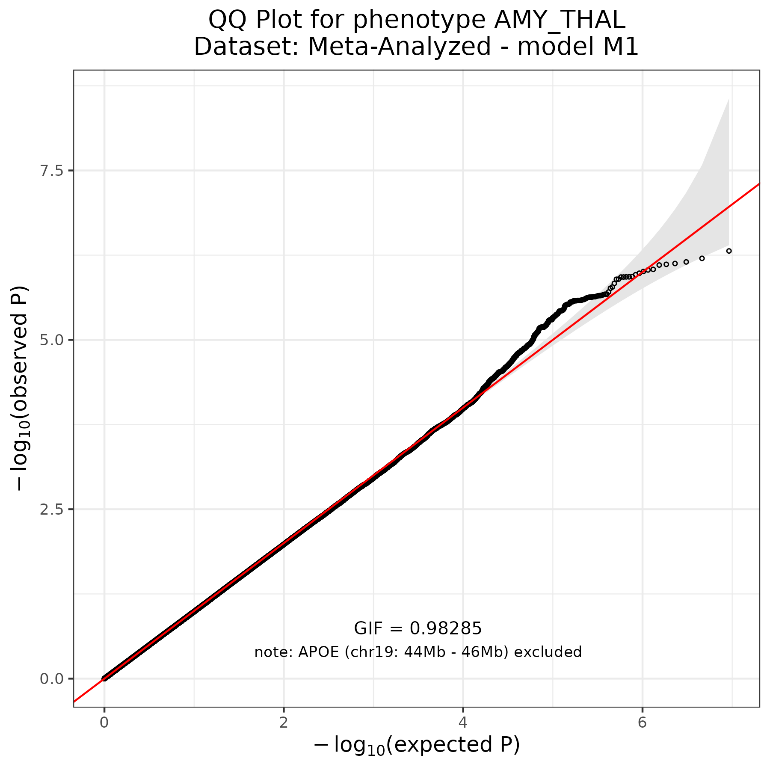

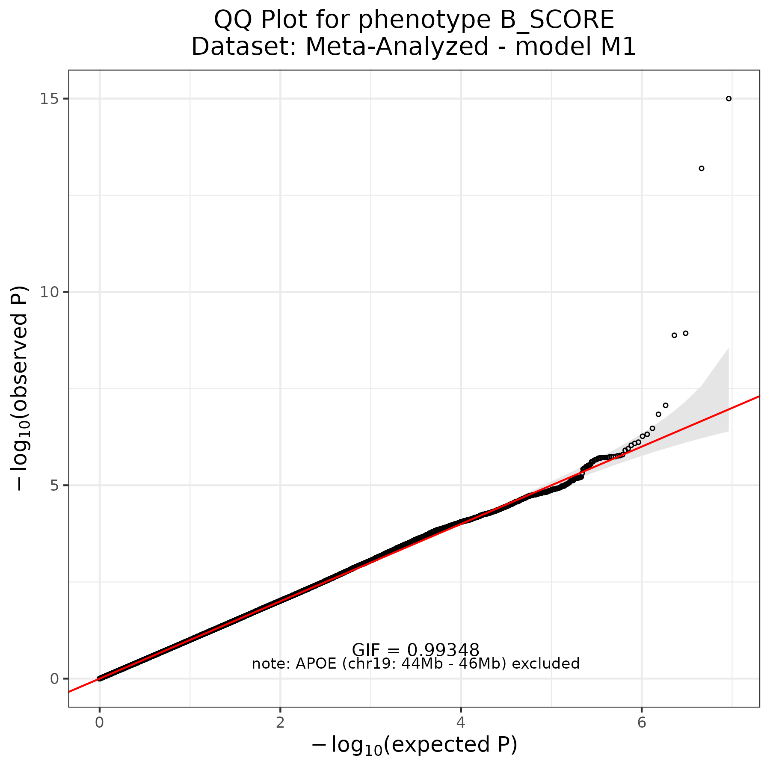

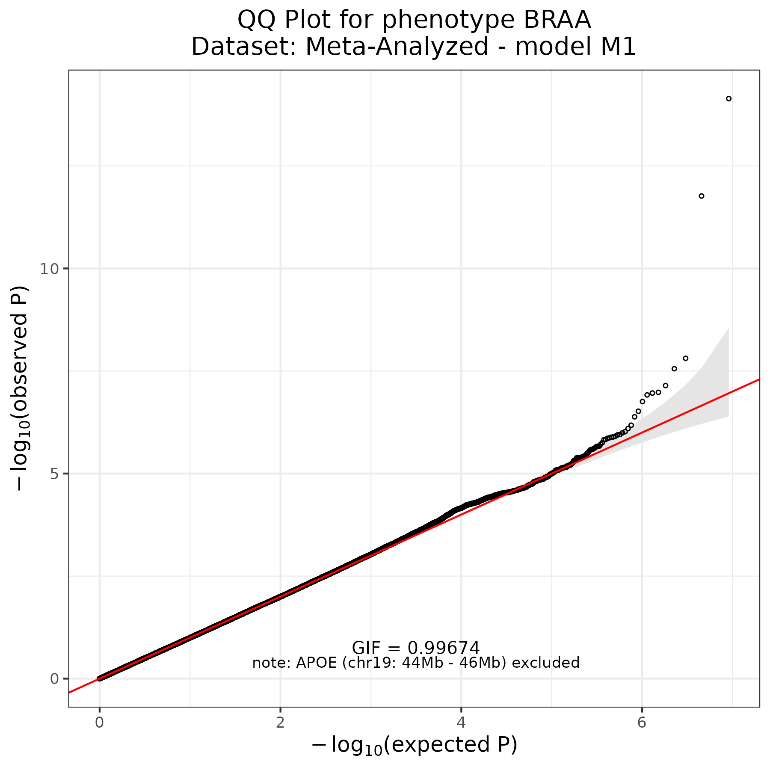

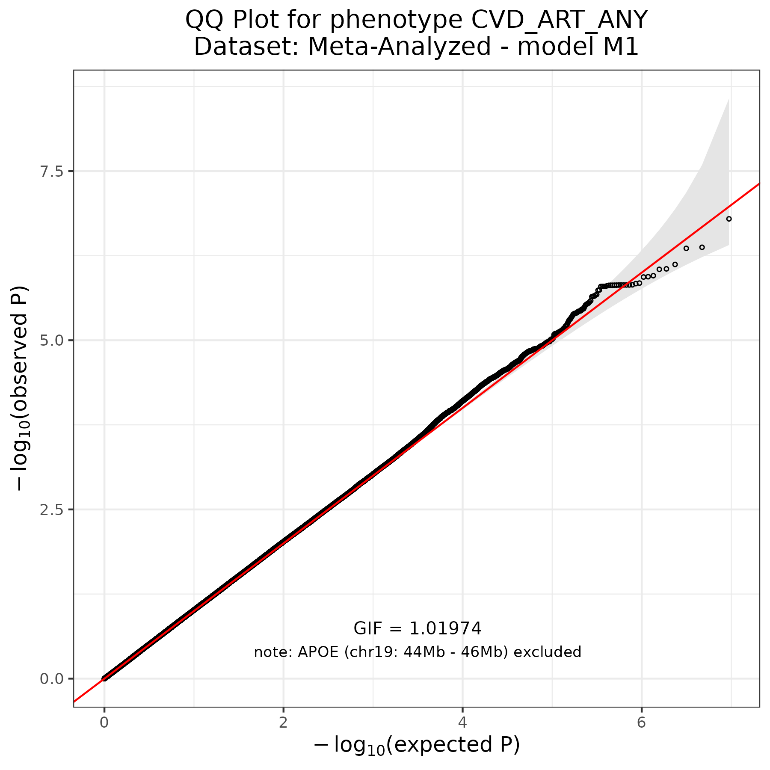

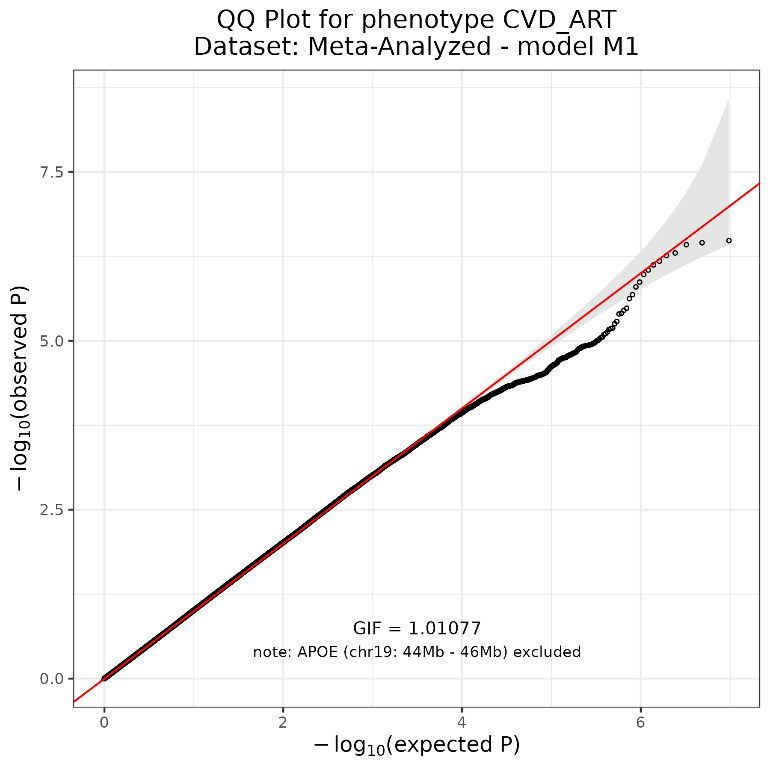

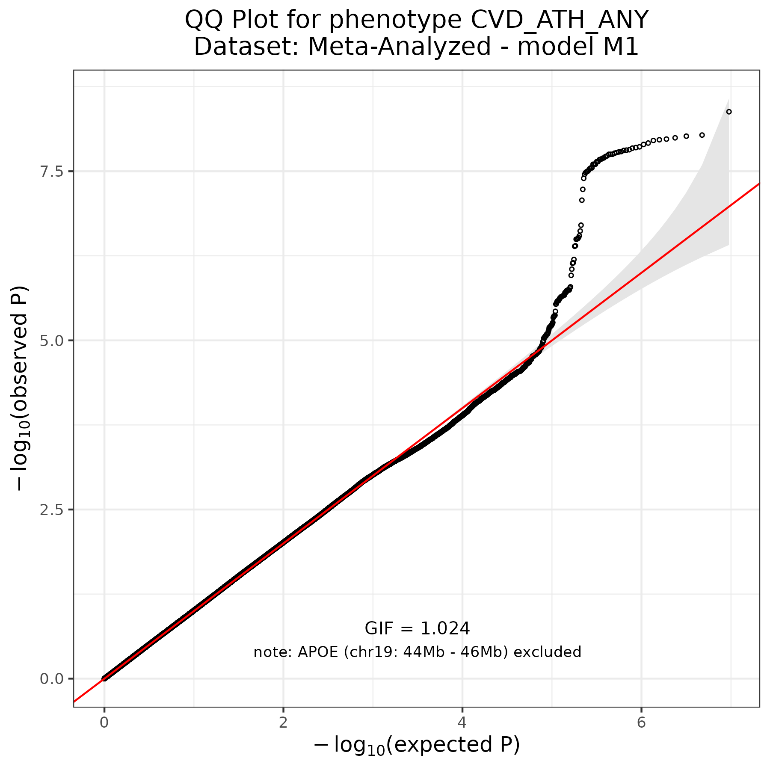

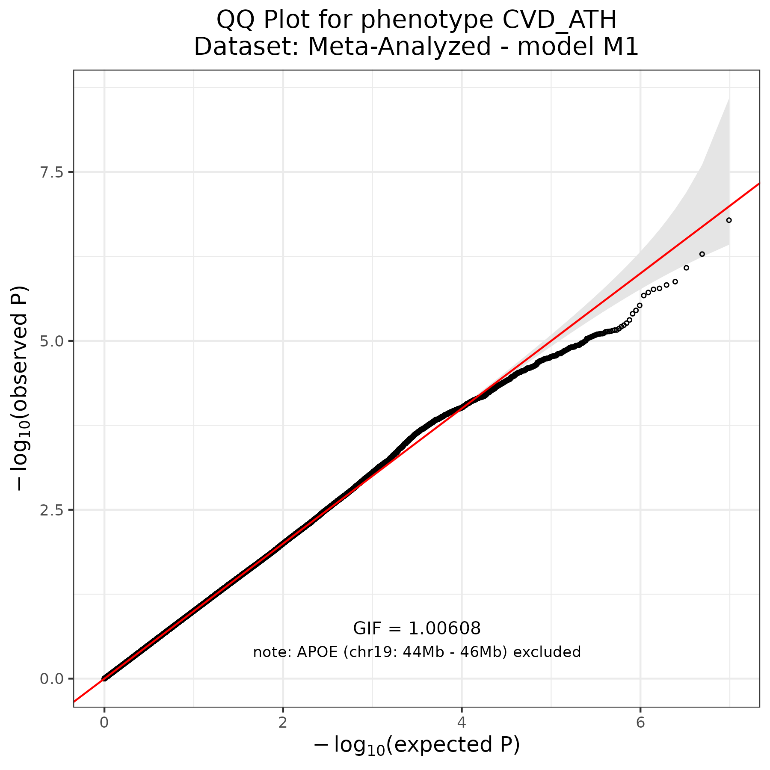

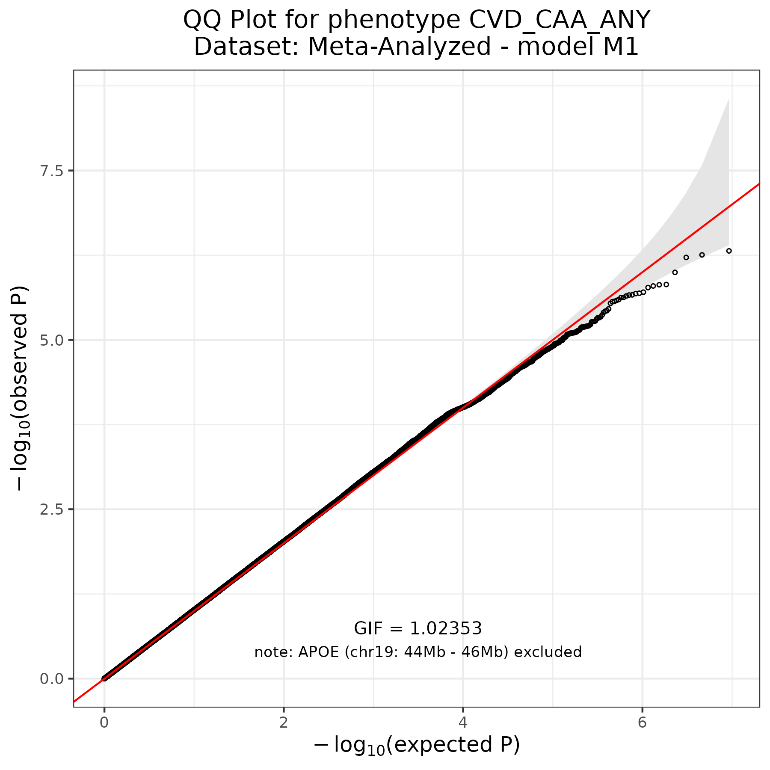

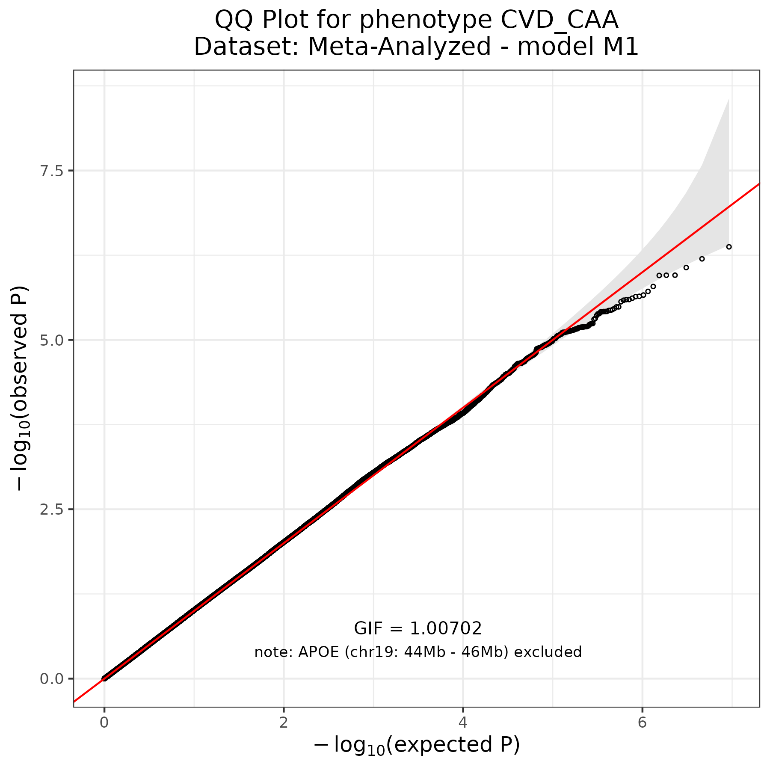

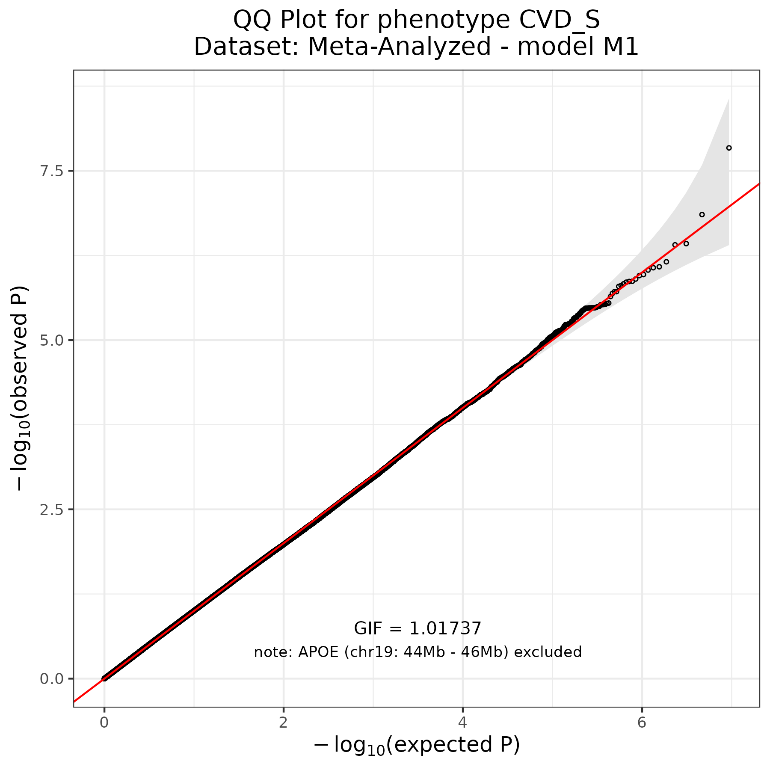

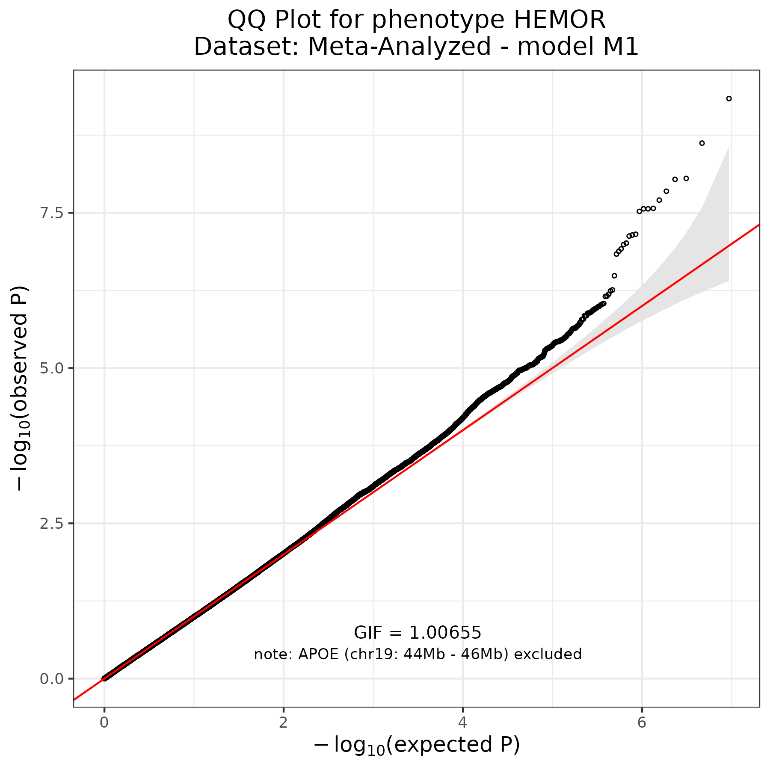

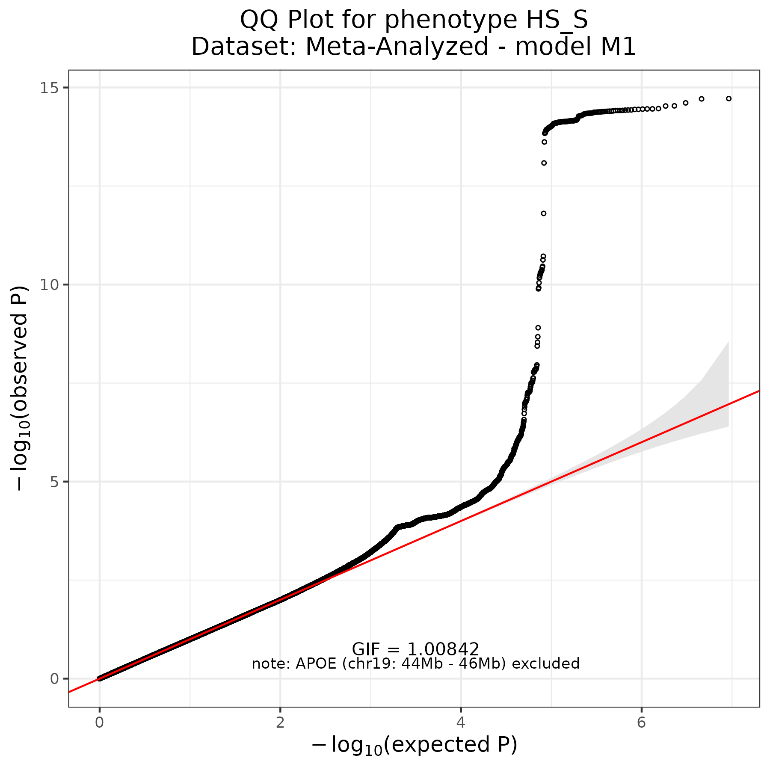

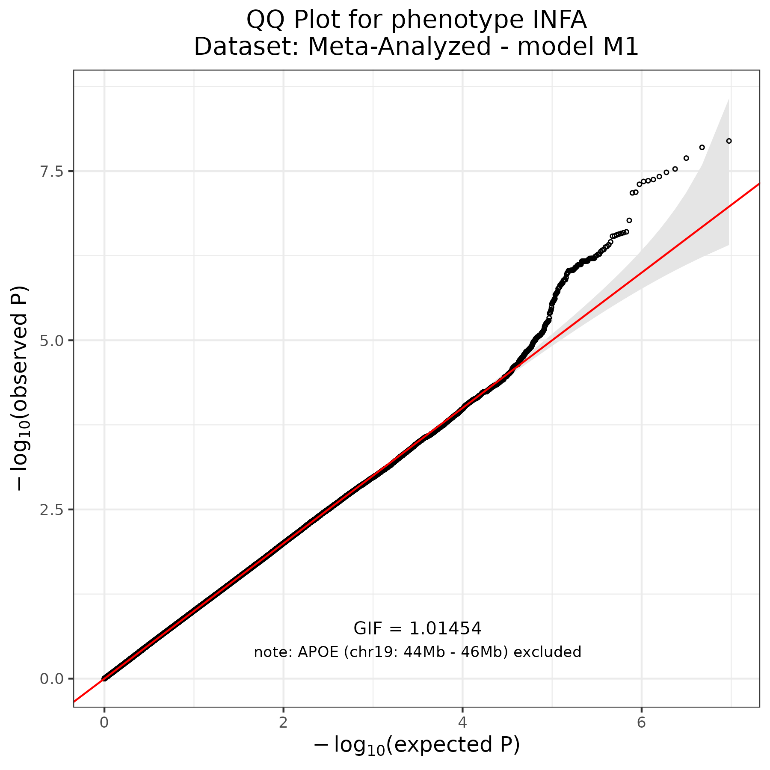

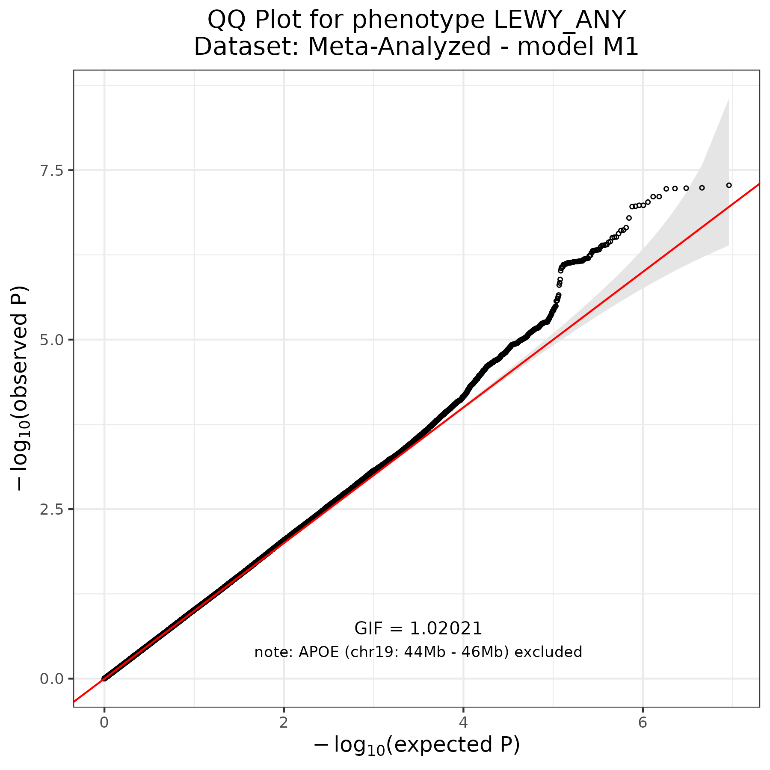

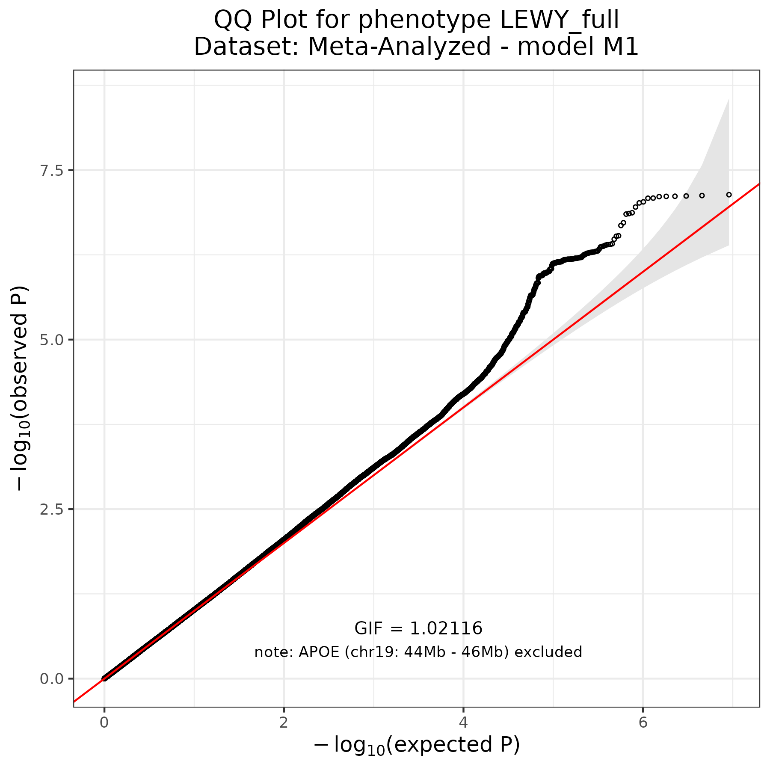

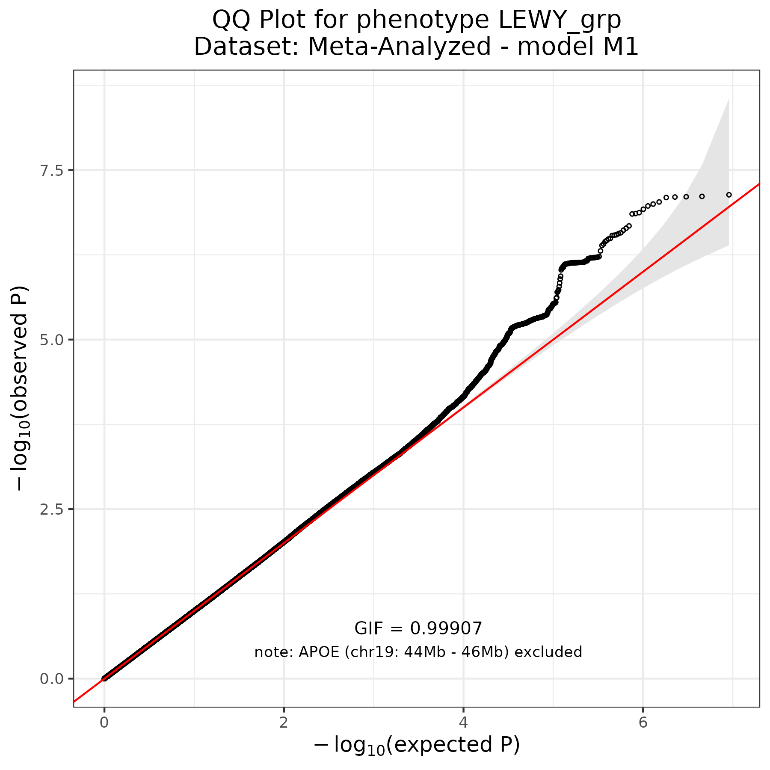

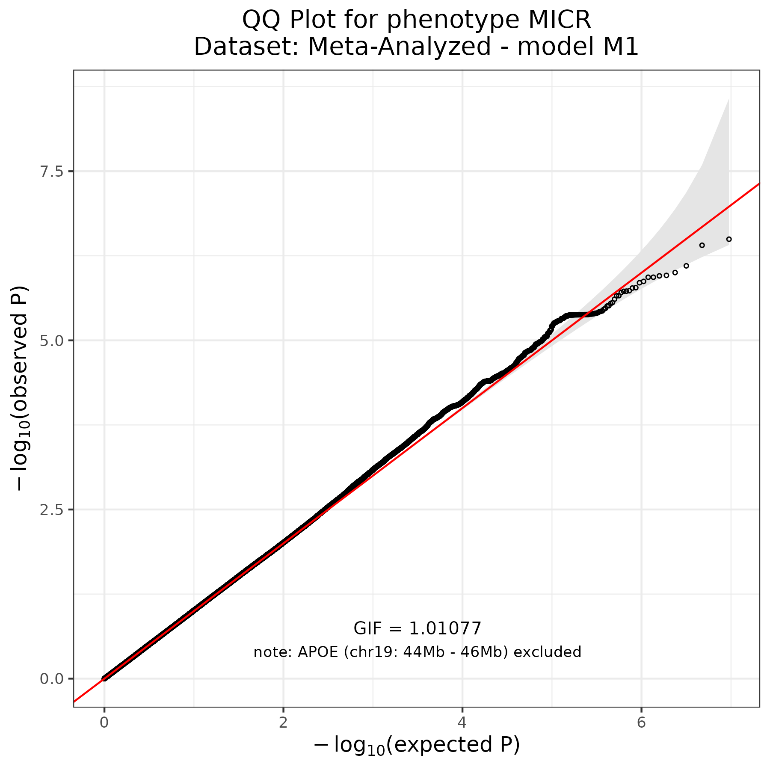

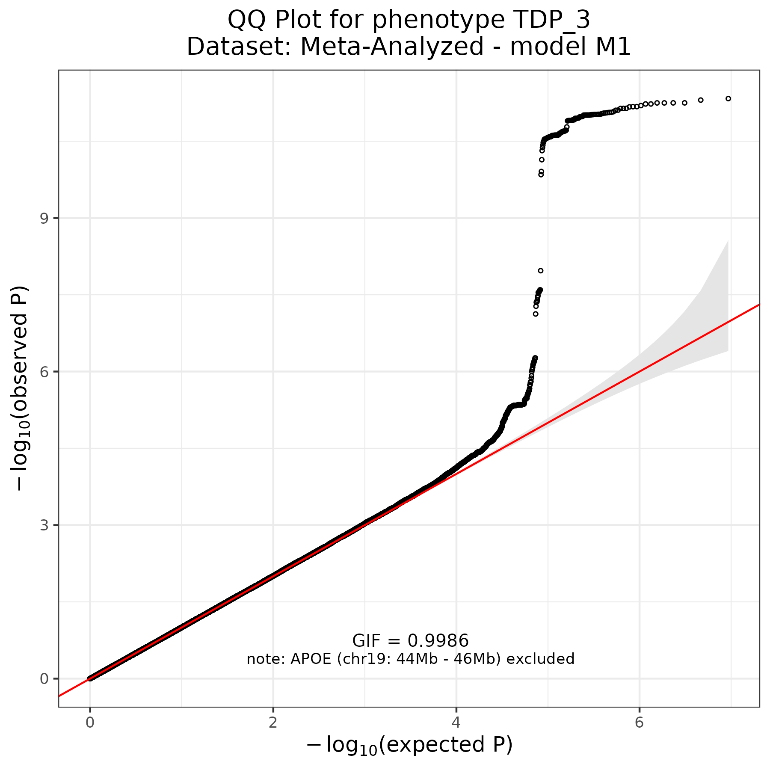

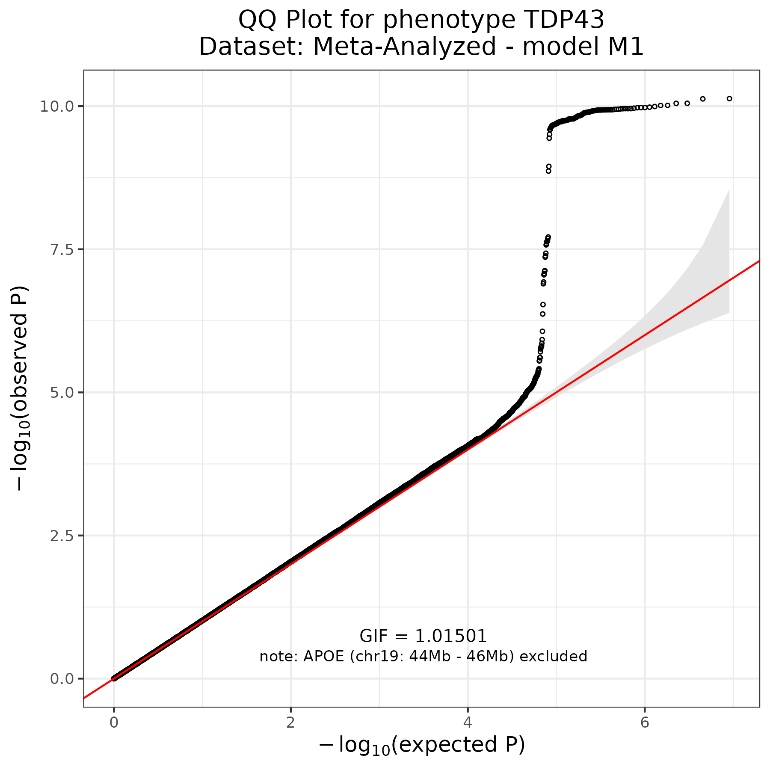

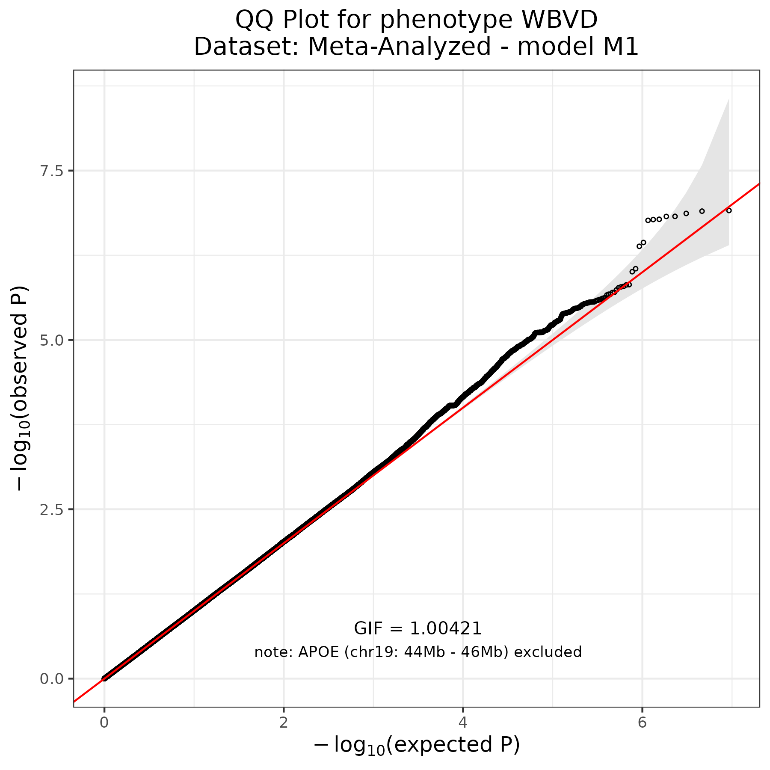

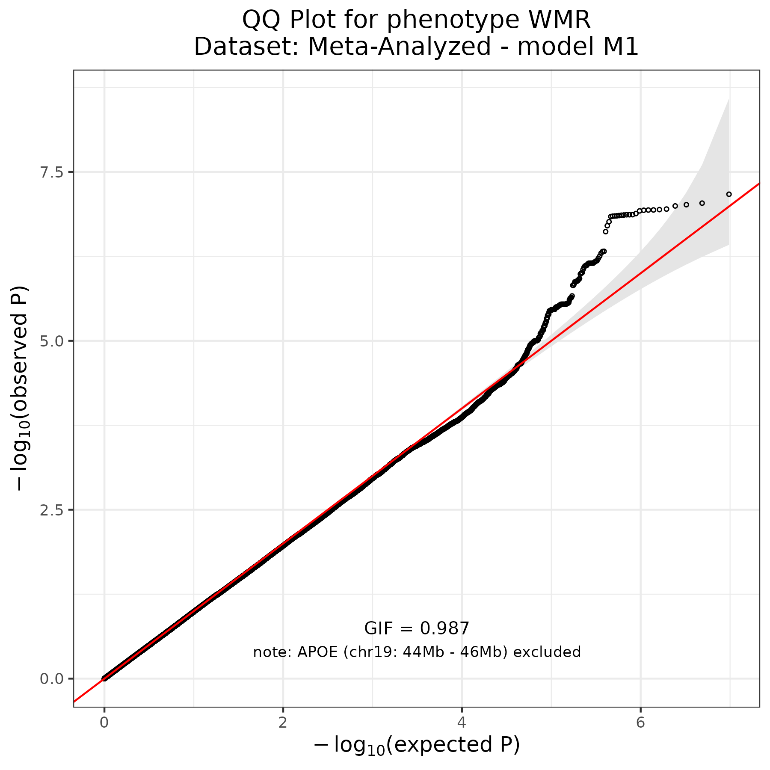

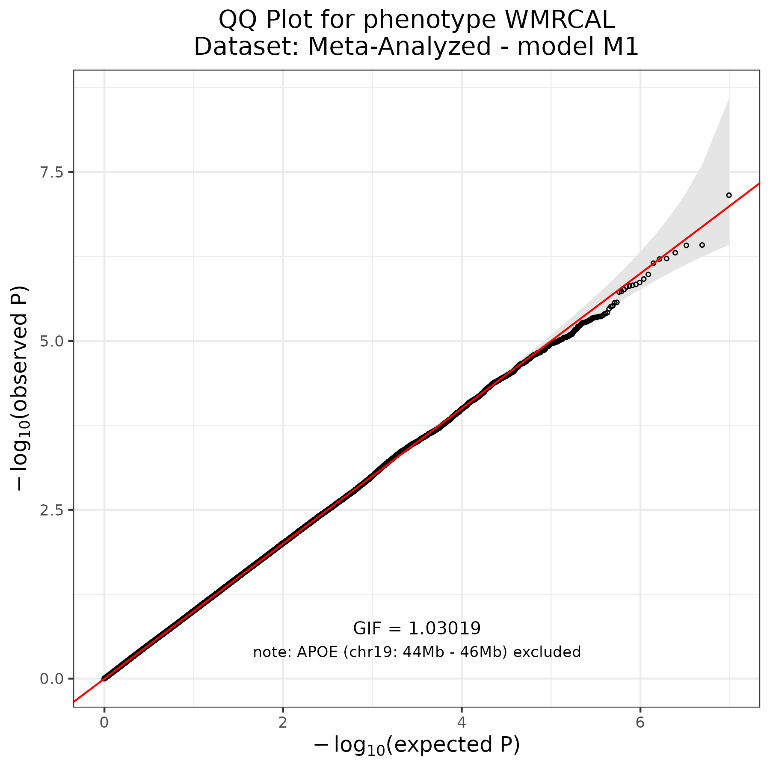

Supplement: S2 Fig — Quantile-quantile plots for each genome-wide association study. λ denotes the genomic inflation factor for the study. (DOCX) [file pgen.1012170.s003.docx]
