# Supplementary material for: Genome wide association study meta-analysis of neuropathologic lesions of Alzheimer’s disease and related dementias in a multi-site autopsy cohort
Source: PLoS Genet. 2026 Jun 29;22(6):e1012170. doi: 10.1371/journal.pgen.1012170 (PMC13340787; doi:10.1371/journal.pgen.1012170)

## Figure S3: P-value by genomic position for A score, B score, and C score

P-values reported on the -log(10) scale. Variants at *APOE* with -log10(p-value) greater than 17 were censored to improve readability.


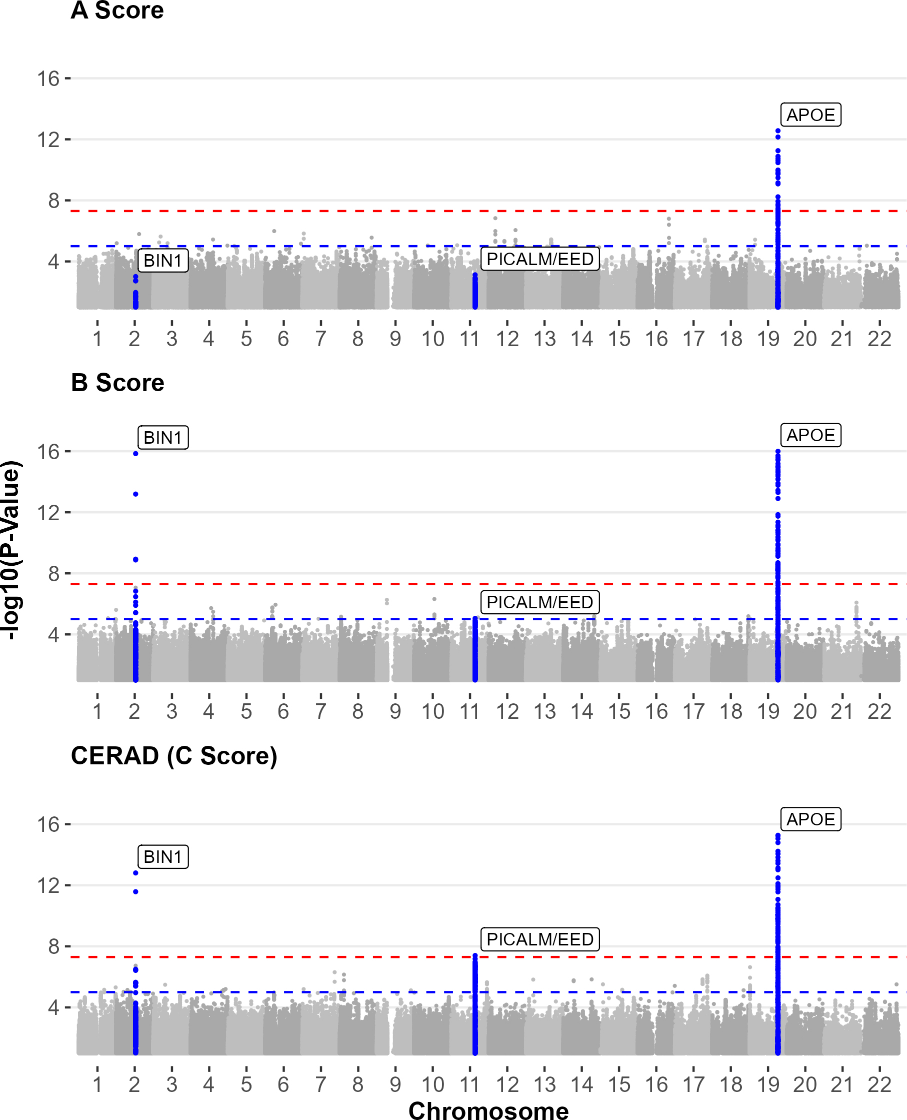

Supplement: S3 Fig — Genome-wide association results for AD hallmark pathologies. P-values reported on the -log(10) scale. Variants at APOE with -log10(p-value) greater than 17 were censored to improve readability. (DOCX) [file pgen.1012170.s004.docx]
