# Supplementary material for: Genome wide association study meta-analysis of neuropathologic lesions of Alzheimer’s disease and related dementias in a multi-site autopsy cohort
Source: PLoS Genet. 2026 Jun 29;22(6):e1012170. doi: 10.1371/journal.pgen.1012170 (PMC13340787; doi:10.1371/journal.pgen.1012170)

## Figure S4: Regional association plot for the *BIN1* locus, for A Score (NP/Thal), B Score (NFT Braak), and C Score (CERAD)


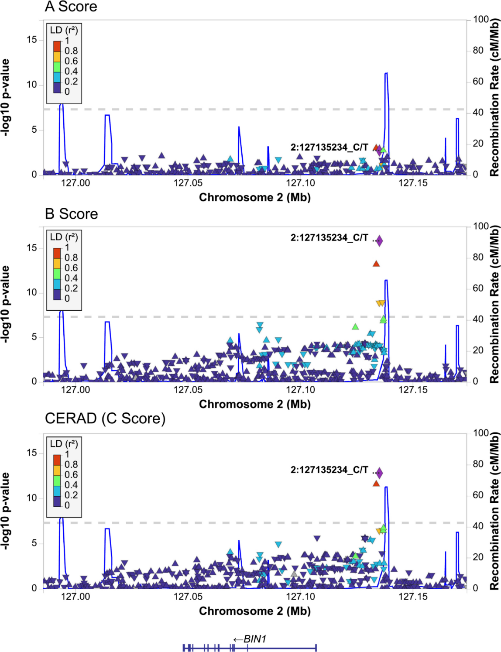

Supplement: S4 Fig — Regional association plots for the BIN1 locus for AD hallmark pathologies. P-values reported on the -log(10) scale. (DOCX) [file pgen.1012170.s005.docx]
