# Supplementary material for: Genome wide association study meta-analysis of neuropathologic lesions of Alzheimer’s disease and related dementias in a multi-site autopsy cohort
Source: PLoS Genet. 2026 Jun 29;22(6):e1012170. doi: 10.1371/journal.pgen.1012170 (PMC13340787; doi:10.1371/journal.pgen.1012170)

## Figure S6: Regional association plots and forest plots for genome-wide significant variants from the amyloid plaque (presence/absence) analysis


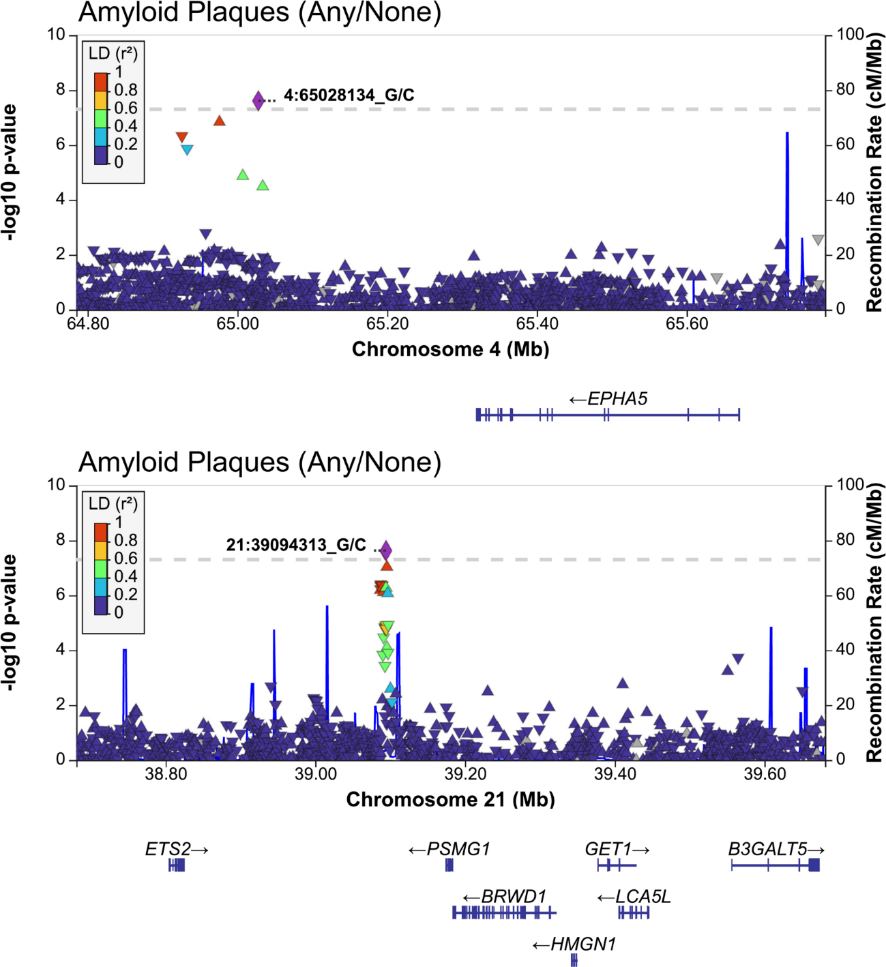


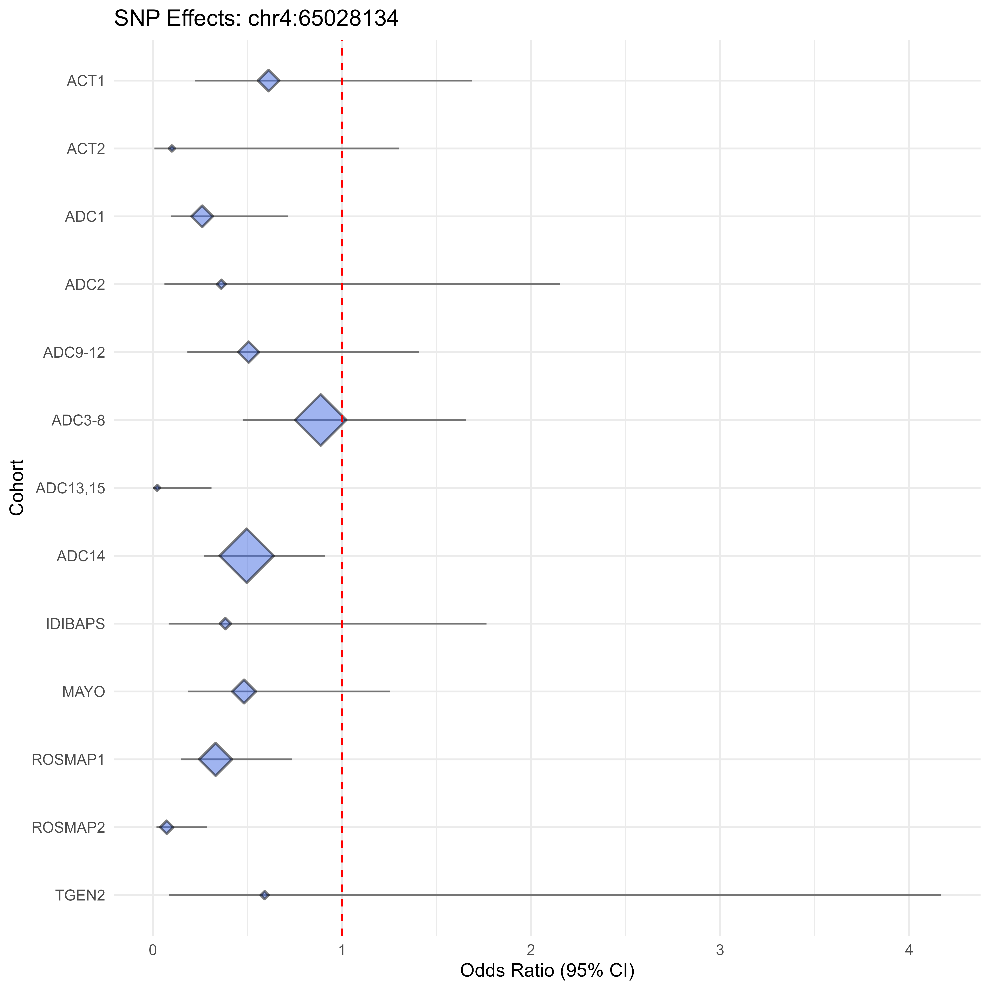

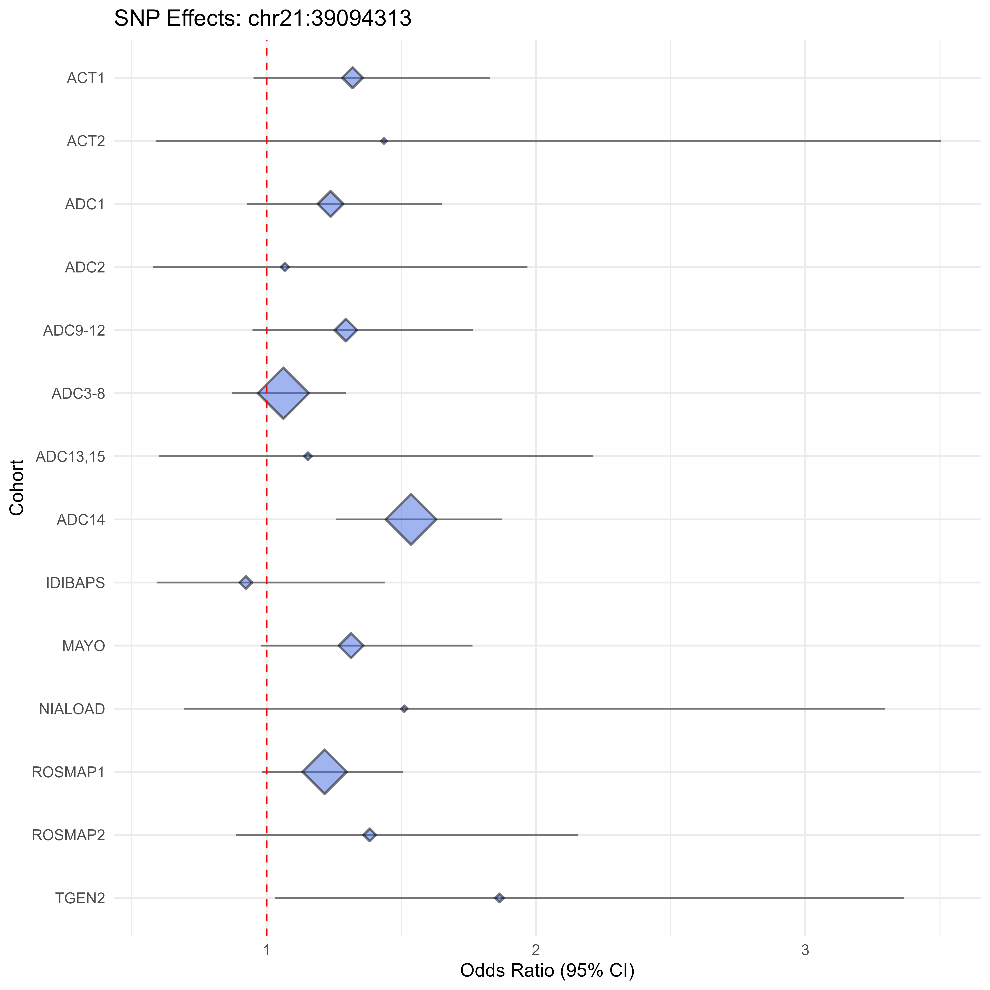

Supplement: S6 Fig — Regional association and forest plots for the EPHA5 and PSMG1 loci for presence/absence of amyloid plaques. P-values reported on the -log(10) scale. (DOCX) [file pgen.1012170.s007.docx]
