# Supplementary material for: Genome wide association study meta-analysis of neuropathologic lesions of Alzheimer’s disease and related dementias in a multi-site autopsy cohort
Source: PLoS Genet. 2026 Jun 29;22(6):e1012170. doi: 10.1371/journal.pgen.1012170 (PMC13340787; doi:10.1371/journal.pgen.1012170)

## Figure S7: P-value by genomic position for association with Thal phase, NFT Braak, and ADNC (ABC) score

P-value was capped at 1e-15. The minimum p-values for *APOE* were: p-value(THAL) = 6.379e-63, p-value(NFT BRAAK) = 8.06e-147, and p-value(ADNC) = 1.916e-55.


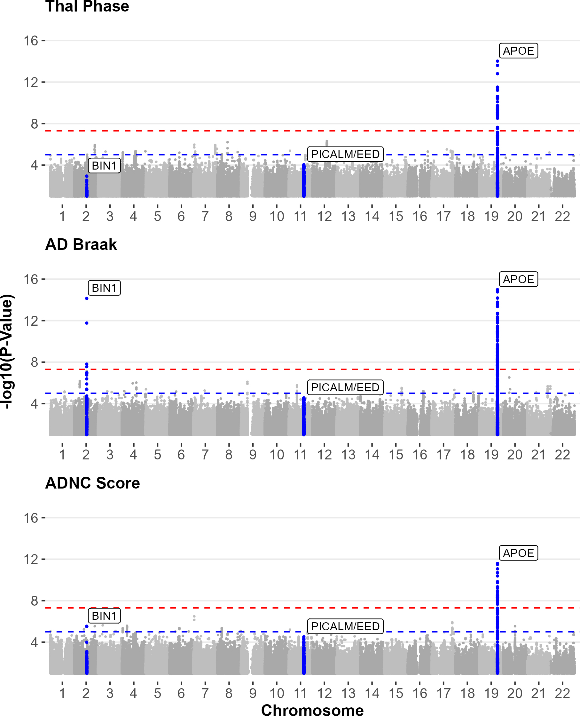

Supplement: S7 Fig — Genome-wide association results for Thal Phase, Braak (NFT), and ADNC composite score. P-value was capped at 1e-15. The minimum p-values for APOE were: p-value(THAL) = 6.379e-63, p-value(NFT BRAAK) = 8.06e-147, and p-value(ADNC) = 1.916e-55. (DOCX) [file pgen.1012170.s008.docx]
