# Supplementary material for: Genome wide association study meta-analysis of neuropathologic lesions of Alzheimer’s disease and related dementias in a multi-site autopsy cohort
Source: PLoS Genet. 2026 Jun 29;22(6):e1012170. doi: 10.1371/journal.pgen.1012170 (PMC13340787; doi:10.1371/journal.pgen.1012170)

## Figure S8: P-value by genomic position for cerebral atherosclerosis (any/none) and cerebrovascular disease (any/none)


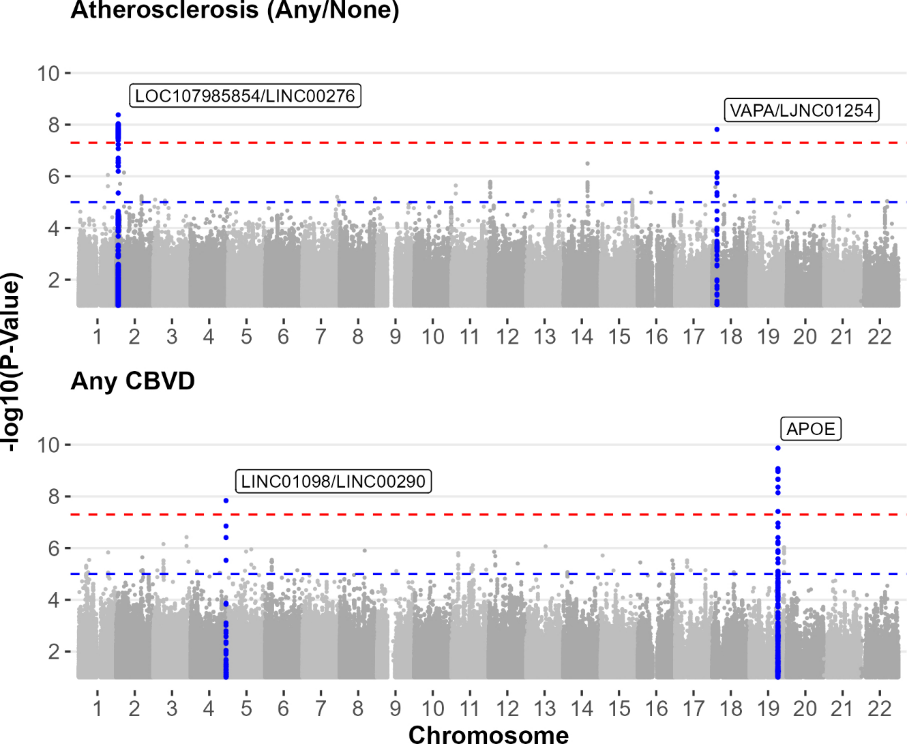

Supplement: S8 Fig — Genome-wide association results for atherosclerosis (any/none) pathology, and cerebrovascular disease (any/none). (DOCX) [file pgen.1012170.s009.docx]
