# Supplementary material for: Genome wide association study meta-analysis of neuropathologic lesions of Alzheimer’s disease and related dementias in a multi-site autopsy cohort
Source: PLoS Genet. 2026 Jun 29;22(6):e1012170. doi: 10.1371/journal.pgen.1012170 (PMC13340787; doi:10.1371/journal.pgen.1012170)

## Figure S9: Regional association plots and forest plots for genome-wide significant variants from cerebral atherosclerosis (any/none) and cerebrovascular disease (any/none) analyses.


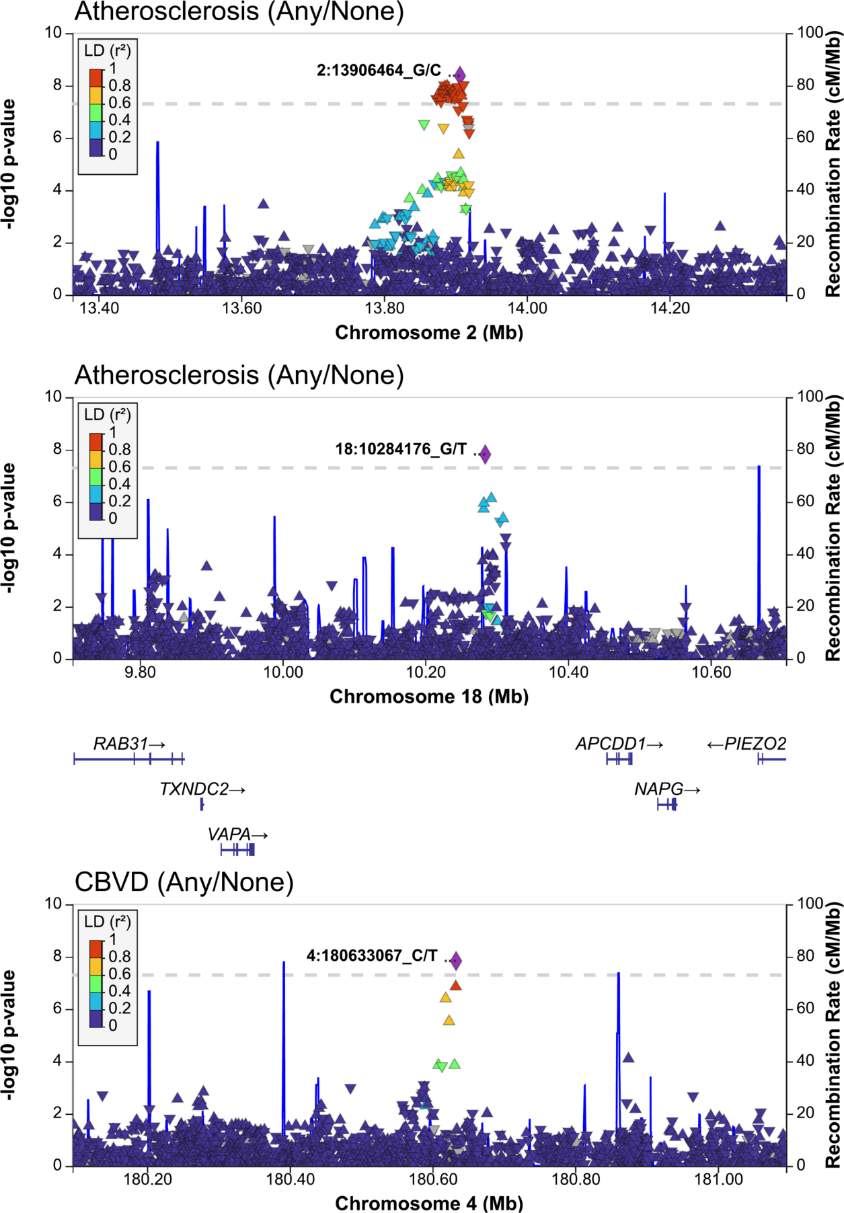


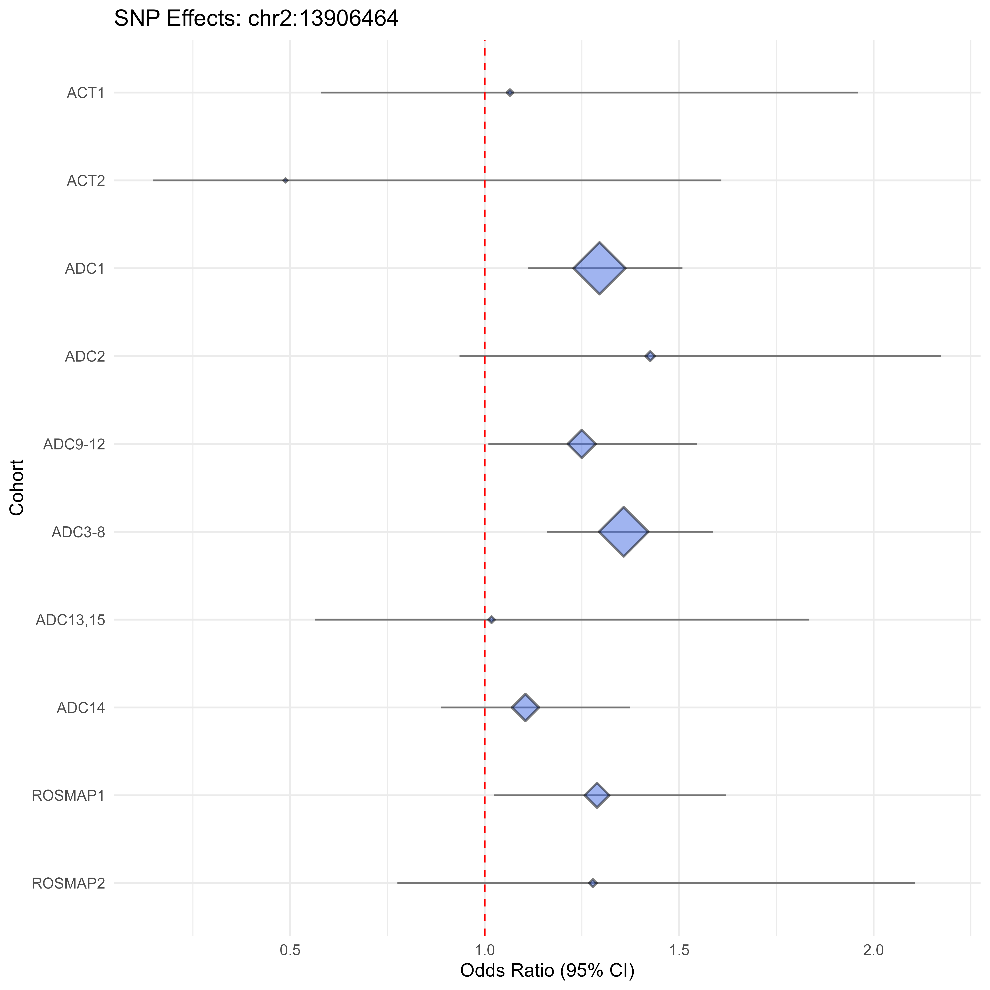


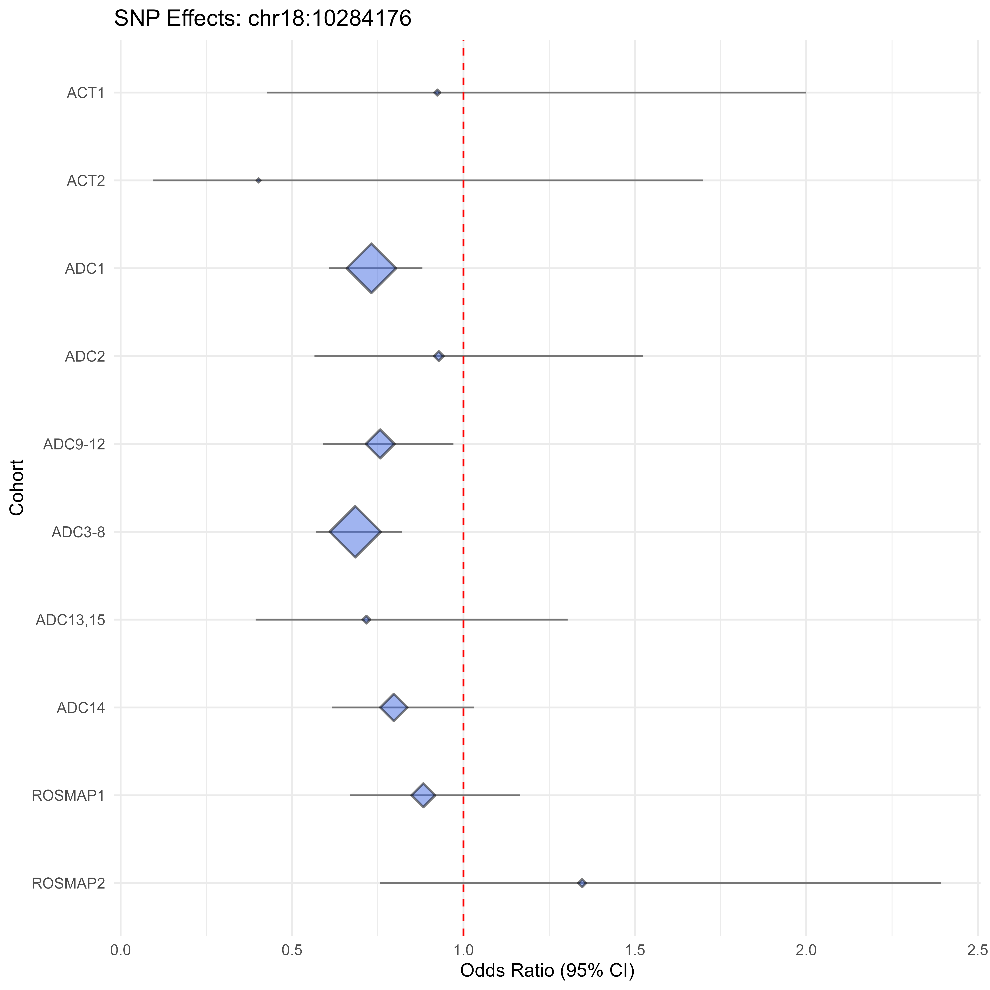


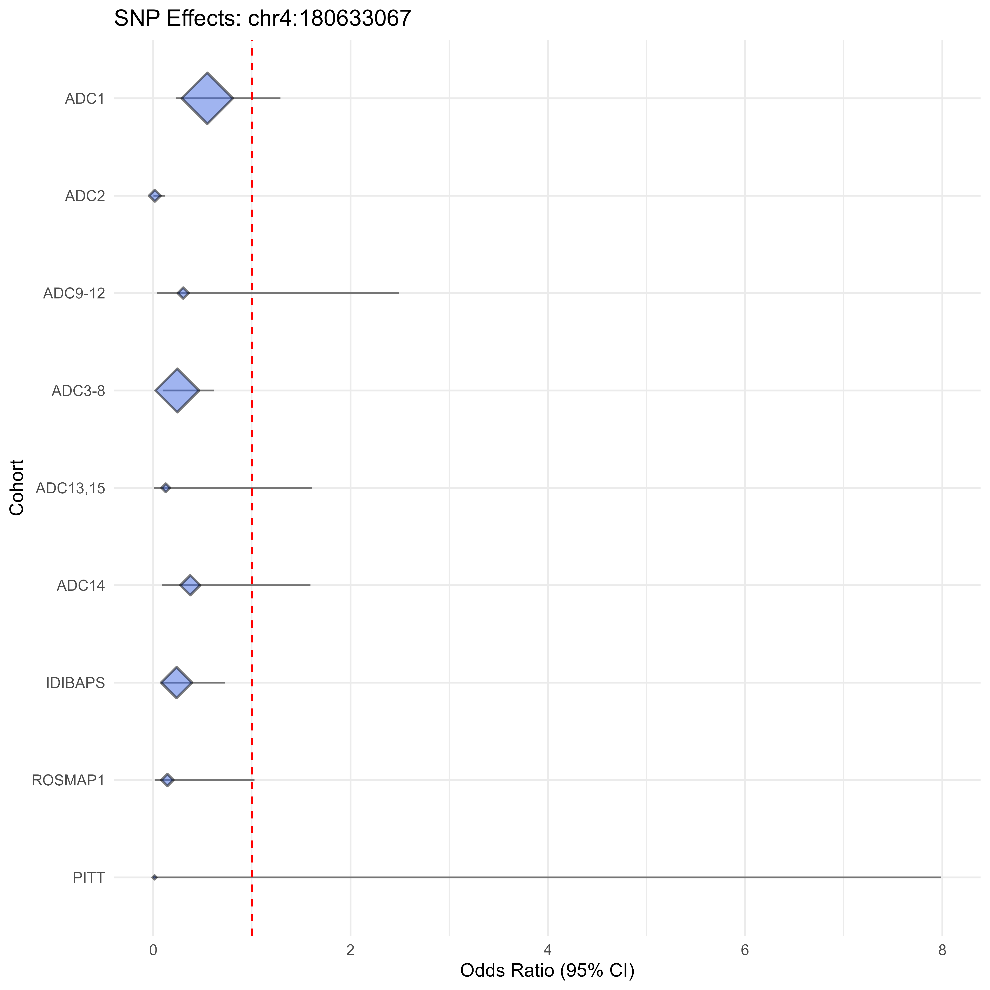

Supplement: S9 Fig — Regional association and forest plots for the significant chromosome 2 locus (atherosclerosis any/none), VAPA (atherosclerosis any/none), and the significant chromosome 4 locus (CBVD any/none). (DOCX) [file pgen.1012170.s010.docx]
