# Supplementary material for: Genome wide association study meta-analysis of neuropathologic lesions of Alzheimer’s disease and related dementias in a multi-site autopsy cohort
Source: PLoS Genet. 2026 Jun 29;22(6):e1012170. doi: 10.1371/journal.pgen.1012170 (PMC13340787; doi:10.1371/journal.pgen.1012170)

## Figure S11: P-value by genomic position for association with infarcts/lacunes analyses, regional association analysis of the *DOCK4* region, and forest plot for the index variant.


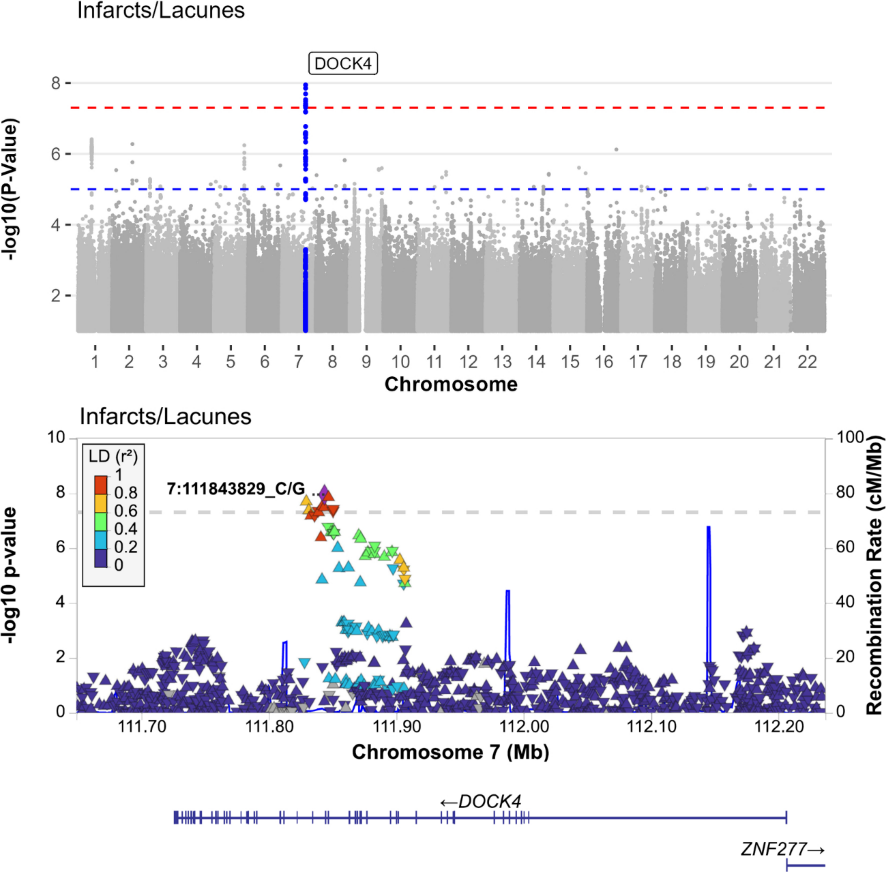


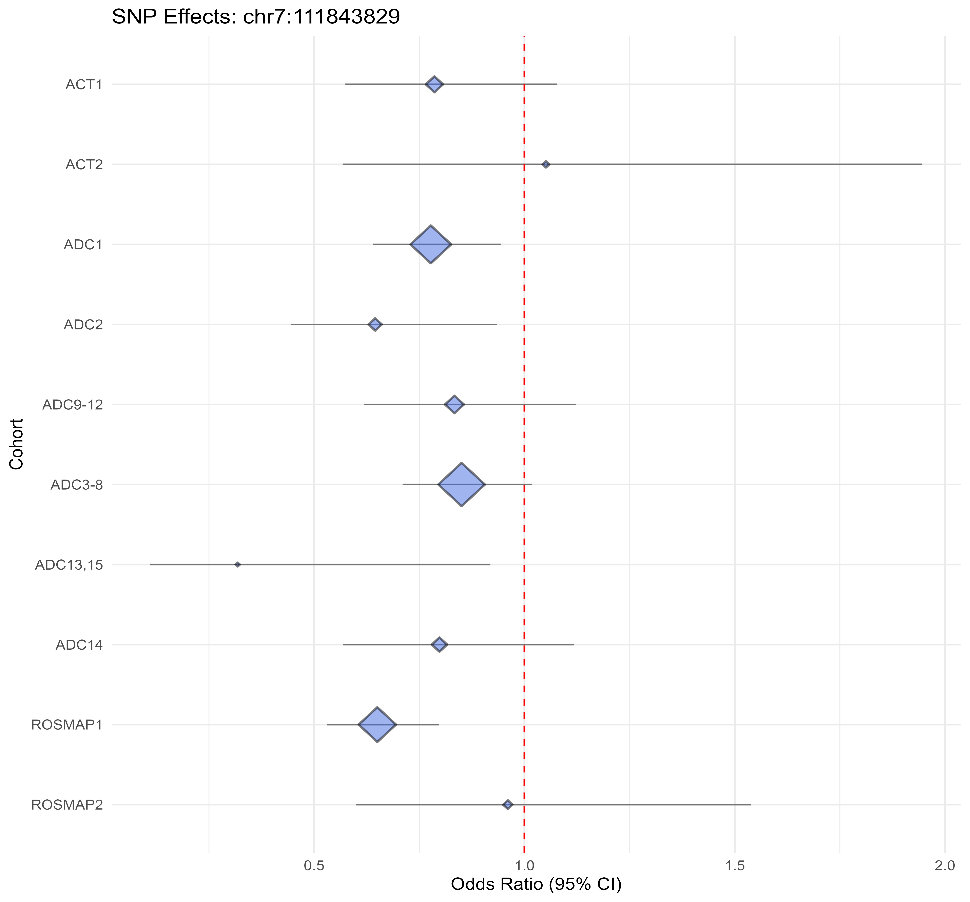

Supplement: S11 Fig — Genome-wide association results for infarcts/lacunes, and regional association plot for the DOCK4 region. P-values reported on the -log(10) scale. (DOCX) [file pgen.1012170.s012.docx]
