# Supplementary material for: Genome wide association study meta-analysis of neuropathologic lesions of Alzheimer’s disease and related dementias in a multi-site autopsy cohort
Source: PLoS Genet. 2026 Jun 29;22(6):e1012170. doi: 10.1371/journal.pgen.1012170 (PMC13340787; doi:10.1371/journal.pgen.1012170)

## Figure S12: P-value by genomic position for association with microinfarcts, hemorrhages, and whole brain vascular disease (WBVD)


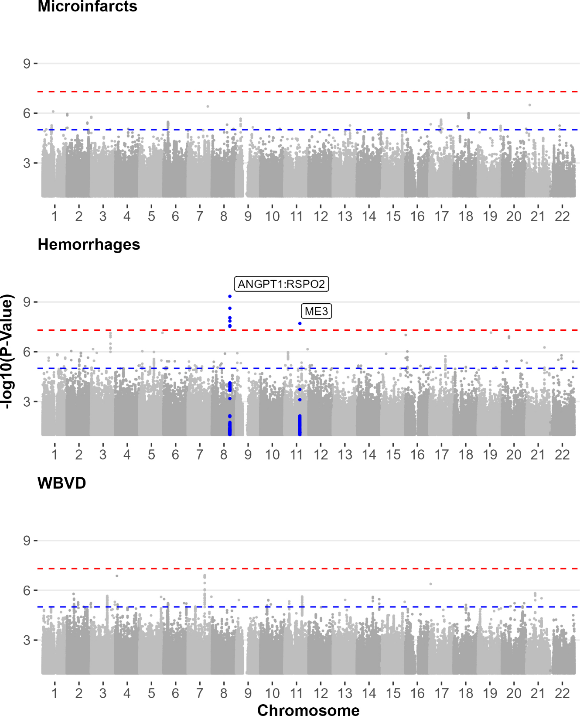

Supplement: S12 Fig — Genome-wide association results for microinfarcts, hemorrhages, and whole brain vascular disease pathologies. P-values reported on the -log(10) scale. (DOCX) [file pgen.1012170.s013.docx]
