# Supplementary material for: Genome wide association study meta-analysis of neuropathologic lesions of Alzheimer’s disease and related dementias in a multi-site autopsy cohort
Source: PLoS Genet. 2026 Jun 29;22(6):e1012170. doi: 10.1371/journal.pgen.1012170 (PMC13340787; doi:10.1371/journal.pgen.1012170)

## Figure S13: P-value by genomic position for association with lewy body (PD Braak) analyses, and regional association analysis of the *SNCA/MMRN1* region.


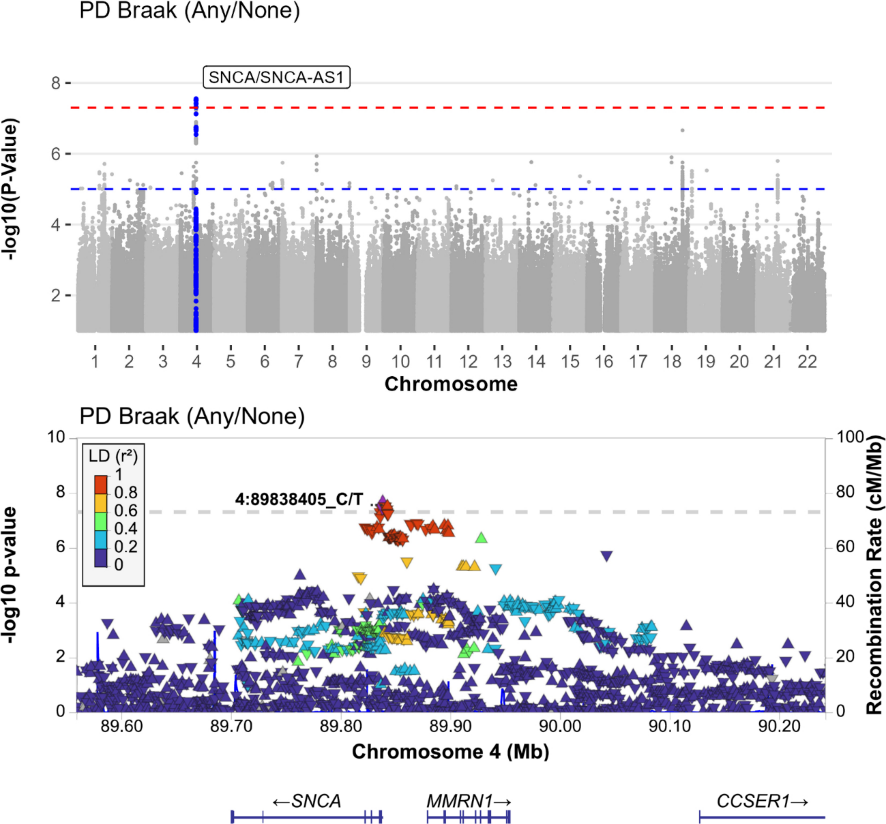

Supplement: S13 Fig — Genome-wide association results for Lewy body pathology (any/none), and regional association plot for the SNCA/MMRN1 region. P-values reported on the -log(10) scale. (DOCX) [file pgen.1012170.s014.docx]
