# Supplementary material for: Genome wide association study meta-analysis of neuropathologic lesions of Alzheimer’s disease and related dementias in a multi-site autopsy cohort
Source: PLoS Genet. 2026 Jun 29;22(6):e1012170. doi: 10.1371/journal.pgen.1012170 (PMC13340787; doi:10.1371/journal.pgen.1012170)

## Figure S14: P-value by genomic position for association with lewy bodies

Model 1 includes age, sex and principal components in the model; model 2 includes *APOE* e4 allele count as well as age, sex, and principal components. Three different parameterizations of lewy bodies were included: PD Braak is the standard 5 category Braak staging for severity of LBD; the “3 cat(egory)” parameterization collapses these into three groups; “Any/None” parameterizes LBD to presence/absence.


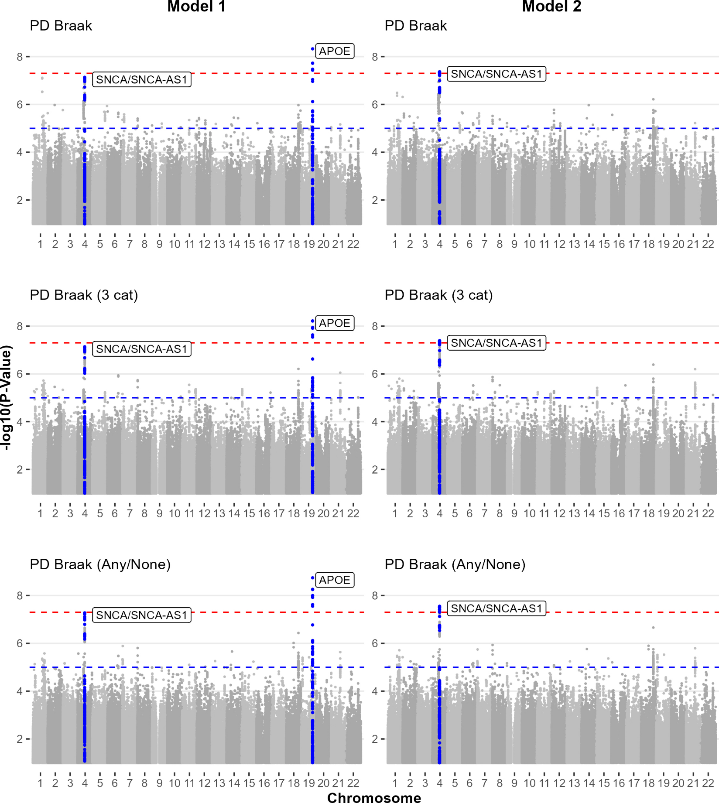

Supplement: S14 Fig — Model 1 includes age, sex and principal components in the model; model 2 includes APOE e4 allele count as well as age, sex, and principal components. Three different parameterizations of lewy bodies were included: PD Braak is the standard 5 category Braak staging for severity of LBD; the “3 cat(egory)” parameterization collapses these into three groups; “Any/None” parameterizes LBD to presence/absence. (DOCX) [file pgen.1012170.s015.docx]
