# Supplementary material for: Genome wide association study meta-analysis of neuropathologic lesions of Alzheimer’s disease and related dementias in a multi-site autopsy cohort
Source: PLoS Genet. 2026 Jun 29;22(6):e1012170. doi: 10.1371/journal.pgen.1012170 (PMC13340787; doi:10.1371/journal.pgen.1012170)

## Figure S15: P-value by genomic position for association with TDP-43 proteinopathy and hippocampal sclerosis


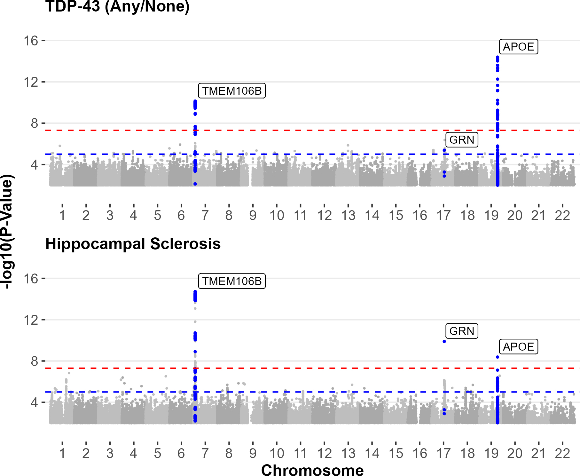

Supplement: S15 Fig — Genome-wide association results for TDP-43 proteinopathy (any/none) and hippocampal sclerosis. P-values reported on the -log(10) scale. (DOCX) [file pgen.1012170.s016.docx]
