# Supplementary material for: Genome wide association study meta-analysis of neuropathologic lesions of Alzheimer’s disease and related dementias in a multi-site autopsy cohort
Source: PLoS Genet. 2026 Jun 29;22(6):e1012170. doi: 10.1371/journal.pgen.1012170 (PMC13340787; doi:10.1371/journal.pgen.1012170)

## Figure S16: Regional association plot for the TMEM106B locus, for TDP-43 proteinopathy and hippocampal sclerosis, and the GRN locus for hippocampal sclerosis.


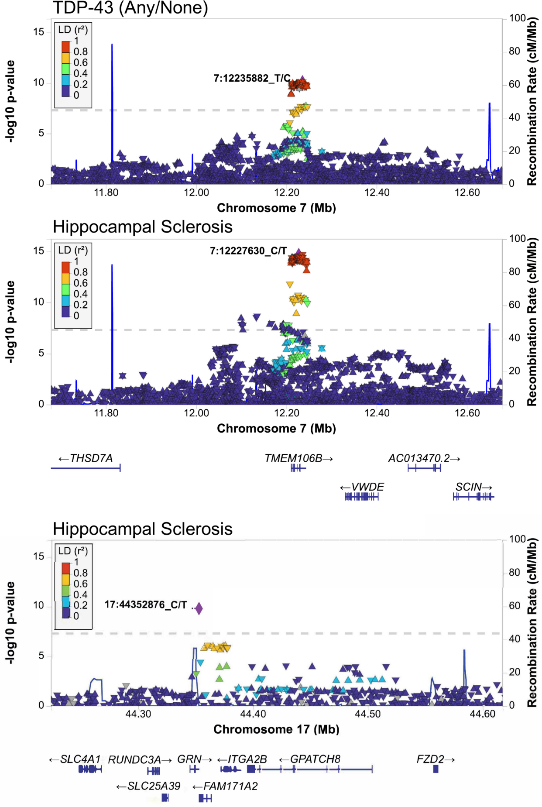

Supplement: S16 Fig — Regional association plot for the TMEM106B locus, for TDP-43 proteinopathy (presence/absence) and hippocampal sclerosis, and the GRN locus for hippocampal sclerosis. (DOCX) [file pgen.1012170.s017.docx]
