# Supplementary material for: Genome wide association study meta-analysis of neuropathologic lesions of Alzheimer’s disease and related dementias in a multi-site autopsy cohort
Source: PLoS Genet. 2026 Jun 29;22(6):e1012170. doi: 10.1371/journal.pgen.1012170 (PMC13340787; doi:10.1371/journal.pgen.1012170)

## Figure S18: Cell-specific heritability enrichment results (ImmGen)

Results from cell-specific heritability enrichment analyses. Y axis denotes -log10(p-value) of the enrichment score. The dashed line denotes nominal association. Study-wide significance by false discovery rate (FDR) would be at -log10(p-value) = 3.03. Variant sets are derived from mouse derived immune gene expression data from the GTEx study, as in Finucane *et al*. See Supplemental Text for more details.


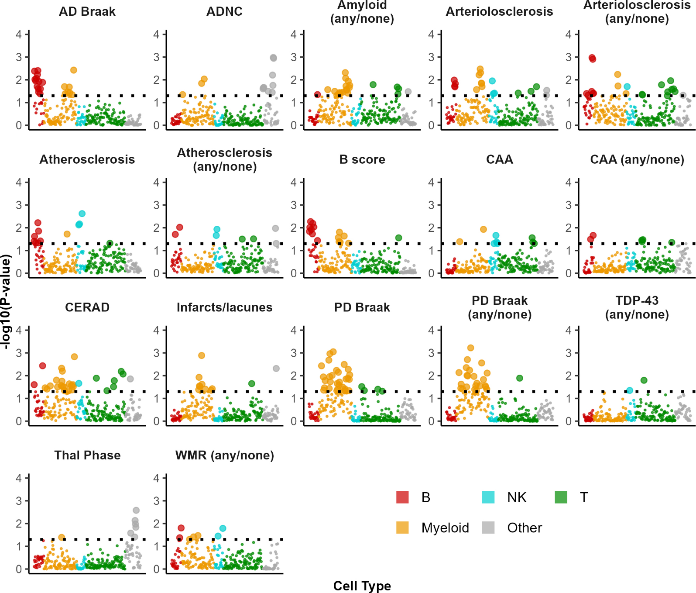

Supplement: S18 Fig — Results from cell-specific heritability enrichment analyses. Y axis denotes -log10(p-value) of the enrichment score. The dashed line denotes nominal association. Study-wide significance by false discovery rate (FDR) would be at -log10(p-value) = 3.03. Variant sets are derived from mouse derived immune gene expression data from the GTEx study, as in Finucane et al. See Supplemental Text for more details. (DOCX) [file pgen.1012170.s019.docx]
